# Huangkui Capsule Ameliorates Renal Fibrosis in a Unilateral Ureteral Obstruction Mouse Model Through TRPC6 Dependent Signaling Pathways

**DOI:** 10.3389/fphar.2020.00996

**Published:** 2020-07-03

**Authors:** Li-fei Gu, Hai-tao Ge, Lei Zhao, Yu-jing Wang, Fan Zhang, Hai-tao Tang, Zheng-yu Cao, Bo-yang Yu, Cheng-zhi Chai

**Affiliations:** ^1^ Jiangsu Provincial Key Laboratory for TCM Evaluation and Translational Research, School of Traditional Pharmacy, China Pharmaceutical University, Nanjing, China; ^2^ Institute of Huanghui, Jiangsu Suzhong Pharmaceutical Group Co., Ltd., Taizhou, China

**Keywords:** Huangkui capsule, kidney fibrosis, TRPC6, unilateral ureteral obstruction model, TGF-β

## Abstract

Renal fibrosis is the final common pathological manifestation of almost all progressive chronic kidney diseases (CKD). Transient receptor potential canonical (TRPC) channels, especially TRPC3/6, were proposed to be essential therapeutic targets for kidney injury. Huangkui capsule (HKC), an important adjuvant therapy for CKD, showed superior efficacy for CKD at stages 1–2 in clinical practice. However, its anti-fibrotic effect and the underlying mechanisms remain to be investigated. In the present study, we evaluated the efficacy of HKC on renal fibrosis in a mouse model of unilateral ureteral obstruction (UUO) and explored the potential underlying mechanism. Administration of HKC by intragastric gavage dose-dependently suppressed UUO-induced kidney injury and tubulointerstitial fibrosis. Similarly, HKC suppressed the expression level of α-smooth muscle actin (α-SMA), increased the expression of E-cadherin, and suppressed the mRNA expression of a plethora of proinflammatory mediators that are necessary for the progression of renal fibrosis. Mechanistically, HKC suppressed both canonical and non-canonical TGF-β signaling pathways in UUO mice as well as the TRPC6/calcineurin A (CnA)/nuclear factor of activated T cells (NFAT) signaling axis. In addition, TRPC6 knockout mice and HKC treated wild type mice displayed comparable protection on UUO-triggered kidney tubulointerstitial injury, interstitial fibrosis, and α-SMA expression. More importantly, HKC had no additional protective effect on UUO-triggered kidney tubulointerstitial injury and interstitial fibrosis in TRPC6 knockout mouse. Further investigation demonstrated that HKC could directly suppress TRPC3/6 channel activities. Considered together, these data demonstrated that the protective effect of HKC on renal injury and interstitial fibrosis is dependent on TRPC6, possibly through direct inhibition of TRPC6 channel activity and indirect suppression of TRPC6 expression.

## Introduction

Renal fibrosis is the common final outcome of almost all progressive chronic kidney diseases (CKD) which affects approximately 10% population for limited options of treatment (Djudjaj and Boor, 2019). Currently, consensus has been reached that tubular cell differentiation and interstitial fibroblast activation play important roles in the process of renal fibrogenesis (Liu, 2011). Calcium ion (Ca^2+^) which is an important signaling molecule is tightly associated with cell differentiation and activation (Clapham, 2007). Transient receptor potential canonical (TRPC) channels are a group of Ca^2+^-permeable, nonselective cation channels. TRPC channels, especially TRPC3/5/6, play critical roles in the development of kidney diseases. Gain-of-function mutations of TRPC6 lead to focal and segmental glomerulosclerosis (FSGS), a common and increasing cause of the end-stage renal disease (Winn et al., 2005; Riehle et al., 2016). Conversely, genetic ablation or pharmacological inhibition of TRPC6 suppresses unilateral ureteral obstruction (UUO)-induced interstitial fibrosis in mice (Wu et al., 2017; Lin et al., 2019). In addition, TRPC6 has also been shown to contribute to the development of experimental pulmonary fibrosis (Hofmann et al., 2017), myofibroblast transdifferentiation and wound healing (Davis et al., 2012). Pharmacological blockade of TRPC6 ortholog, TRPC3, inhibits fibroblast proliferation and myofibroblast differentiation (Saliba et al., 2015) and genetic ablation of TRPC3 attenuates UUO-induced renal fibrosis in mice (Wu et al., 2017). Although TRPC5 has been shown to alter podocyte function, inconsistent findings were reported. Genetic ablation or pharmacologic inhibition of TRPC5 has been reported to benefit kidney filter dynamics by balancing podocyte cytoskeletal remodeling (Schaldecker et al., 2013; Zhou et al., 2017). However, another group showed that the overexpression or activation of the TRPC5 ion channels cannot cause kidney barrier injury or aggravate such injury under pathologic conditions (Wang et al., 2017). In addition, there is no evidence that TRPC5 channels play roles in the progression of renal interstitial fibrosis.

*Abelmoschus manihot* (L.) Medik. (*A. manihot*), which belongs to Malvaceae family, is a medical plant widely used to treat inflammatory disease since ancient China (Rubiang-Yalambing et al., 2016). In 1999, based on the effectiveness on CKD in clinical practice, a formulation (Huangkui Capsule, HKC) of ethanol extract of *A. manihot* flowers was approved by China’s State Food and Drug Administration under category III of traditional Chinese medicine for chronic nephritis treatment (Chen et al., 2016). A multicenter, randomized, controlled clinical trial demonstrates that HKC displays superior anti-proteinuria efficacy than losartan in patients with CKD at stages 1-2 (Carney, 2014; Zhang et al., 2014). In type II diabetic patients, HKC significantly decreases the levels of proteinuria and serum creatinine (Scr) (Chen et al., 2015). Currently, HKC has been used as an important adjuvant therapy for CKD (Zhang et al., 2014). Pharmacological studies have reported the protective effect of HKC against renal injury in diabetic nephropathy and adriamycin-induced renal injury animal models. HKC decreases albuminuria, attenuates early glomerular pathology and renal tubular epithelial–mesenchymal transition in the diabetic nephropathy animal model (Mao et al., 2015; Ge et al., 2016; Kim et al., 2018; Wu et al., 2018; Han et al., 2019). Similarly, in an adriamycin-induced renal injury murine model, HKC attenuates kidney inflammation and glomerular injury, likely through inhibition of reactive oxygen species (ROS)-mitogen-activated protein kinase (MAPK) signaling pathway (Tu et al., 2013; Mao et al., 2015; Li et al., 2019).

Chemical and pharmacological investigation has revealed that flavonoids are the main bioactive chemical constitutes of HKC to improve diabetic nephropathy (Lai et al., 2009). Pharmacokinetic studies demonstrate that the flavonoids are the main compounds detected in the blood and kidney tissue suggesting that the flavonoids are the potential active components (Lai et al., 2007; Xue et al., 2011). In human kidney-2 cells, the flavonoids in HKC, including quercetin, isoquercitrin, hyperoside, gossypetin-8-*O*-*β*-*D*-glucuronide and quercetin-3′-*O*-glucoside, prevents epithelial to mesenchymal transition (Cai et al., 2017). However, the molecular mechanism of anti-fibrotic effects of HKC remains to be established.

UUO surgery is the widely used renal fibrosis model, which displays characteristic tubular cell injury, interstitial inflammation, and fibrosis, recapitulating the pathology observed from both irreversible acute kidney injury and CKD (Chevalier et al., 2009; Ucero et al., 2014). In the present study, we evaluated the efficacy of HKC against renal fibrosis in UUO mice and the possible involvement of TRPC channels on HKC renal protection and anti-fibrotic effects. We demonstrate that HKC displays anti-fibrotic effect in a UUO mouse model with better efficacy than losartan. Further mechanistic studies demonstrate that the anti-fibrotic effect of HKC is through TRPC6 dependent pathway.

## Materials and Methods

### Drugs and Chemicals

HKC was purchased from Jiangsu SuZhong Pharmaceutical Group Co., Ltd. (16061806, Taizhou, Jiangsu, China). The content of hyperoside in HKC determined by HPLC was 1.97%, which conformed to the standard of flowers of *A. manihot* in the Chinese Pharmacopoeia (2015 edition, hyperoside ≥0.50%). Losartan potassium was purchased from Hangzhou MSD Pharmaceutical Co., Ltd. (Hangzhou, Zhejiang, China). Scr (C011-2-1) and blood urea nitrogen (C013-2-1, BUN) assay kits were purchased from Nanjing Jiancheng Biotech Co., Ltd (Nanjing, Jiangsu, China). Hydroxyproline ELISA quantification kit was purchased from JinYiBai Biological Technology Co., Ltd. (Nanjing, Jiangsu, China). The primary antibodies including anti-α-smooth muscle actin (ab32575, anti-α-SMA) and anti-calcineurin A (ab109412, anti-CnA) were purchased from Abcam Inc. (Cambridge, MA, USA) while the antibodies against E-cadherin (3195), phospho-p38 (4511), c-Jun N-terminal kinase (9252, JNK), phospho-JNK (4668), extracellular regulated protein kinases 1/2 (4695, ERK1/2), phospho-ERK1/2 (4370), smad2 (5339), and smad3 (9523) were from Cell Signaling Technology (Beverly, MA, USA). Anti-p38 antibody (14061-1-AP) was obtained from Proteintech Group, Inc. (Chicago, IL, USA). Anti-TRPC6 antibody (ACC-017) was purchased from Alomone Labs Ltd. (Jerusalem, Israel). Anti-nuclear factor of activated T cells (DF6446, NFAT) antibody was obtained from Affbiotech Company (Cincinnati, OH, USA). Anti-GAPDH antibody (MB001) and anti-β-Tubulin antibody (MB8025) were purchased from Bioworld Technology (Nanjing, Jiangsu, China). IRDye 680RD- and 800CW-labeled secondary antibodies were purchased from LI−COR Biotechnology (Lincoln, NE, USA). Alexa Fluor^®^ 488 goat anti-rabbit secondary antibody (A11034) was purchased from Invitrogen (Carlsbad, CA, USA). 4,6-diamidino-2-phenylindole (C1005, DAPI) was purchased from Beyotime Biotech. (Nanjing, Jiangsu, China). Total RNA Extraction Reagent, HiScript Q RT SuperMix for qPCR and ChamQ SYBR qPCR Master Mix (Low ROX Premixed) were purchased from Vazyme Biotech (Nanjing, Jiangsu, China).

The reference standards of quercetin-3-*O*-robinobioside (PubChem CID: 10371536), hyperoside (PubChem CID: 5281643), isoquercitrin (PubChem CID: 5280804), gossypetin-8-*O*-*β*-*D*-glucuropyranoside (PubChem CID: 133613102), myricetin (PubChem CID: 5281672), quercetin-3′-*O*-*β*-*D*-glucoside (PubChem CID: 5280804) and quercetin (PubChem CID: 5280343) (purity > 98%) were purchased from Shanghai Yuanye Biotechnology Co., Ltd. (Shanghai, China).

### HPLC Analysis of the Chemical Constitutes of HKC

The chemical constitutes of HKC were analyzed on a ZORBAX SB-C18 reverse-phase column (4.6 mm × 250 mm, 5 μm, Agilent Technologies Inc., Santa Clara, CA, USA) performed on an Agilent 1260 Series HPLC system and the absorptions were monitored by a VW detector at the wavelength of 254 nm. The column was eluted with a gradient concentration of acetonitrile with trifluoroacetic acid (TFA) at a flow rate of 1 mL/min using the following program. Solvent A: 0.1% TFA in ddH_2_O, solvent B: acetonitrile, 0–5 min, 6% B; 5–30 min, 6–17% B; 30–40 min, 17% B; 40–60 min, 17–40% B.

### Generation of TRPC6 Knockout Mice

TRPC6 knockout (KO) mice were generated in Animal Core Facility of Nanjing Medical University based on CRISPR-Cas9 approach combined with a double-nicking strategy (Ran et al., 2013). Exon 7 of *Trpc6*, located on chromosome 9, was chosen to disrupt the *Trpc6* gene. *Trpc6* gene disruption was confirmed by genotyping using nested PCR analysis with genomic DNA as the template and two sets of primers as follows: 5′-TCCCCTTATTCAAGTCAGAATATACTACA-3′, and 5′-GGGAGGTATTTGTCATGTAATCTGACTC-3′ for the first step; 5′-ATACTACACACACTTGAGAAGTTCTTCAGA-3′, and 5′-TTGGGAAGGTTCCTTTATGCTAGT-3′ for the second step. Predicted PCR products were 827 bp for wild type (WT) mice and 590 bp for the TRPC6 KO mice.

### Preparation of HKC Solution

HKC solutions were prepared as described previously (Mao et al., 2015). Animal dose (1.5 g/kg/day) was obtained according to the daily dose of HKC (7.5 g/day) in clinical practice. Two additional lower doses (0.5 and 0.15g/kg/day) were also selected to explore the dose-dependency of HKC protective effects on UUO-induced kidney injury. For animal experiments, HKC content was dissolved in ddH_2_O to obtain 0.015, 0.05, and 0.15 g/mL HKC solutions. For patch clam experiment, HKC content was dissolved in DMSO to obtain a stock solution of 300 mg/mL. The stock solution was then diluted with bath solution (see patch clamp section) to final concentrations of 300, 100, 30, and 10 μg/mL.

### Cell Culture and Treatment

HEK-293 cells stably expressing mouse TRPC3 or TRPC6 channels were the same lines as described previously (Yang et al., 2019). Cells were plated in 35-mm dishes at a density of 1,000 cells/dish and cultured in Dulbecco’s modified eagle medium supplemented with 10% fetal bovine serum, 10 mM HEPES, 100 U/mL penicillin, 0.1 mg/mL streptomycin, and 200 μg/mL G418 at 37°C in an incubator with 5% CO_2_ and 95% humidity. Cells were cultured for 6–12 h before patch clamp experiments.

### UUO Model and Treatment

All the animal care and experimental protocols were approved by the Animal Ethics Committee of China Pharmaceutical University according to Guidelines of the National Institutes of Health for the Care and Use of Laboratory Animals (Protocol No. 220182328). UUO surgery was performed as described previously (Gu et al., 2018). To evaluate the efficacy of HKC on renal fibrosis, a total of 70 male C57BL/6 mice (20–23 g) were randomly divided into 7 groups: (1) sham group, (2) sham plus HKC (1.5 g/kg), (3) UUO mice receiving ddH_2_O, (4)-(6) UUO mice receiving low (0.15 g/kg), medium (0.5 g/kg), and high dose (1.5 g/kg) of HKC, respectively, and (7) UUO mice receiving losartan (10 mg/kg/day). All the drugs were administrated by intragastric gavage (*i.g.*, 10 mL/kg) for 7 consecutive days right after surgery. The HKC dose (1.5 g/kg/day) was equal to the clinical dosage. To verify whether HKC protective effect was through TRPC6 channel, HKC (1.5 g/kg) was administered *i.g.* for 7 consecutive days in wild type (WT) and TRPC6 KO mice after UUO. Blood samples were collected from ophthalmic veins on day 8. Left kidneys were dissected and half of each left kidney was kept in 10% formalin solution and sectioned for histological examination while the remaining part was frozen into liquid nitrogen for protein and total RNA extraction.

### Serum Creatinine, Blood Urea Nitrogen and Hydroxyproline Assay

Scr and BUN levels were quantitatively examined using Scr and BUN assay kits according to the manufacture’s instruction. Serum hydroxyproline was determined by an ELISA assay using mouse hydroxyproline quantification kit according to the manufacture’s instruction.

### Hematoxylin and Eosin and Masson’s Trichrome Staining

Four kidneys were randomly chosen in each group and were fixed with 10% formalin, dehydrated, embedded in paraffin, and sliced to a thickness of 5 µm. Sections were stained with hematoxylin and eosin (H & E) or Masson’s trichrome and the pictures were digitized using Nanozoomer whole slide scanner (Hamamatsu Photonics, Hamamatsu, Japan). The kidney injury score was assessed by determining the tubular atrophy and degeneration, renal papillary necrosis, interstitial inflammation, and fibrous hyperplasia according to the scoring criteria described previously (Debelle et al., 2002).

### Immunofluorescence Staining

Kidney sections were blocked with 5% bovine serum albumin, followed by incubation with primary antibodies against α-SMA (1:200), or E-cadherin (1:200) overnight at 4°C. After washing with PBS for 3 ×, an Alexa Fluor^®^ 488 conjugated goat anti-rabbit secondary antibody (1:300) was added and incubated for 1.5 h at RT. After washing with PBS for 3 ×, a concentration of 2 μg/mL DAPI was added and incubated for 10 min to visualize the nuclei. All sections were digitized using a digital CMOS camera (Hamamatsu photonics) attached to a Leica DMI8 invert fluorescence microscope (Leica Microsystems, Wetzlar, Germany).

### Western Blotting

Western blotting was performed as described previously (Shen et al., 2019). Briefly, dissected kidneys were homogenized in RIPA buffer containing protease inhibitor cocktail. After quantification of the protein concentration using BCA kit, equal amount of proteins (40 μg) were loaded to wells of SDS-polyacrylamide gels (10%) and separated by electrophoresis. The proteins were then transferred onto nitrocellulose membranes by electroblotting. After blocking with 5% BSA, primary antibodies against α-SMA, E-cadherin, p38, phospho-p38, JNK, phospho-JNK, ERK1/2, phospho-ERK1/2, smad2, smad3, TRPC6, CnA, NFAT (1:1000), or GAPDH (1:10,000) were added. After incubation overnight at 4 ˚C, the membranes were incubated with the IRDye (680RD or 800CW)-labeled secondary antibodies (1:10,000 dilution) for 1 h at RT and scanned for densitometry with a LI−COR Odyssey Infrared Imaging System (LI−COR Biotechnology). Densitometry was carried out using LI-COR Odyssey Infrared Imaging System application software (Ver. 2.1, LI-COR Biotechnology).

### Reverse-Transcription PCR

Total RNA was extracted using TRIzol reagent and reversed-transcribed to cDNA using HiScript Q RT SuperMix. Quantitative real-time PCR (qPCR) was carried out using ChamQ SYBR qPCR Master Mix (Low ROX Premixed) in QuantStudio 3 Real-Time PCR System (Thermo Fisher Scientific). GAPDH was used as the internal control to normalize the mRNA levels of interleukins (*Il-1β*, *Il-12*, *Il-6*), monocyte chemoattractant protein-1 (*Mcp-1*), transforming growth factor (*Tgf*)*-β*, matrix metalloproteinase-12 (*Mmp12*), vascular cell adhesion protein 1 (*Vcam1*), *α-Sma* and *E-cadherin*. The primers used were listed in **Table S1**.

### Patch Clamp Electrophysiology

Whole cell patch-clam recording was conducted at RT using an EPC-10 amplifier (HEKA, Pfalz, Germany) controlled by PatchMaster software (HEKA) as described previously (Yang et al., 2019). Recording electrodes were pulled from borosilicate glass using P-1000 Micropipette Puller (Sutter Instrument, Novato, CA, USA) to 2–3 MΩ when filled with pipette solution containing (in mM): 140 CsCl, 1 MgCl_2_, 5 EGTA, and 10 HEPES, pH = 7.2. The bath solution contains (in mM): 140 NaCl, 5 KCl, 2 CaCl_2_, 1 MgCl_2_, 10 glucose, and 10 HEPES, pH = 7.4. Cells were clamped at 0 mV and currents elicited by a voltage ramp from −100 mV to +100 mV every 1 s were recorded at 5 kHz. Compounds were delivered through a press-driven multichannel system (ALA Scientific Instruments, Farmingdale, NY, USA) to the cell being recorded.

### Data Analysis

All data points represent the mean ± SEM. The concentration–response curves were generated using GraphPad software fitted by a non-linear logistic equation (Ver 7.0, GraphPad Software Inc., San Diego, CA, USA). Statistical significance between groups was calculated using one-way ANOVA followed by post-hoc Dunnett’s multiple comparisons. A *P* value below 0.05 was considered to be statistically significant.

## Results

### Analysis of the Chemical Constitutes of HKC

An HPLC analysis resolved seven main peaks of HKC (**Figure S1A**). By comparing the retention time (*Rt*) with flavonoid standards (**Figure S1B**), these peaks were unambiguously confirmed to be quercetin-3-*O*-robinobioside, hyperoside, isoquercitrin, gossypetin-8-*O*-*β*-*D*-glucuropyranoside, myricetin, quercetin-3′-*O*-*β*-*D*-glucoside, and quercetin (**Figure S1**). Quantitative analysis demonstrate that the contents of quercetin-3-*O*-robinobioside, hyperoside, isoquercitrin, gossypetin-8-*O*-*β*-*D*-glucuropyranoside, myricetin, quercetin- 3′-*O*-*β*-*D*-glucoside and quercetin were 0.52%, 1.97%, 1.16%, 2.07%, 0.21%, 1.08%, and 0.15%, respectively.

### HKC Ameliorated UUO-Induced Kidney Injury and Renal Fibrosis in Mice

The level of serum BUN, but not Scr was significantly higher in mice subjected to UUO compared to sham group (9.17 ± 0.23 mmol/L *vs.* 6.45 ± 0.17 mmol/L, *P* < 0.01, n = 10) (**Table S2**). Similarly, UUO mice displayed significant higher level (17.13 ± 0.37 μg/L) of hydroxyproline compared to sham control (14.33 ± 0.62 μg/L) (*P* < 0.01, n = 10). Oral administration of HKC (1.5 g/kg/d) for seven consecutive days, had marginal effect on the basal levels of Scr, BUN, and hydroxyproline (*P* > 0.05, n = 10) (**Table S2**). However, administration of HKC at doses of 0.15 g/kg, 0.5 g/kg and 1.5 g/kg in UUO mice decreased the BUN levels to 7.60 ± 0.30, 7.44 ± 0.22, and 7.38 ± 0.19 mmol/L, respectively and reduced the hydroxyproline levels to 16.55 ± 0.68, 15.61 ± 0.44 and 14.97 ± 0.57 μg/L, respectively, in UUO mice (**Table S2**). As the positive control, losartan (10 mg/kg) decreased serum concentration of BUN (*P* < 0.01, n = 10) but not hydroxyproline concentration (**Table S2**).

**Figure 1:**
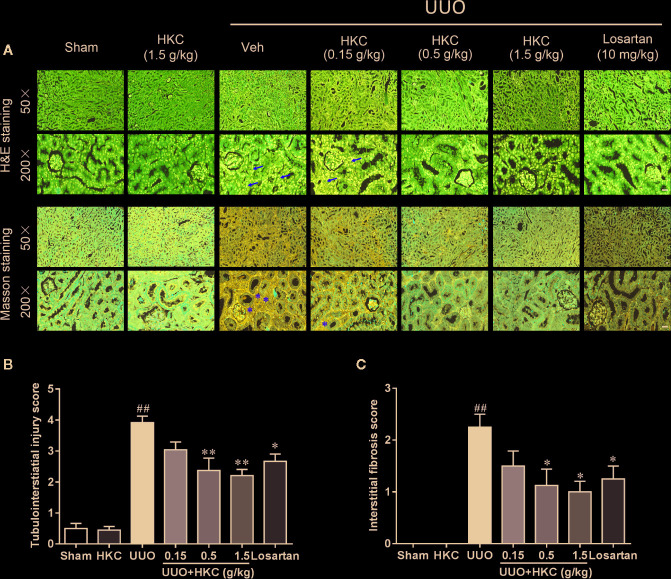
HKC ameliorates kidney injury in UUO mice. **(A)** Representative images of H&E staining (upper two panels) and Masson’s trichrome staining (lower two panels) of kidney sections from sham and UUO mice with different treatment. Yellow arrowheads indicated the inflammatory cell infiltration of the kidney tissues. Yellow asterisks indicated the fibrotic tissues. Scale bar = 100 μm. **(B)** Quantification of tubulointerstiatial injury score in H & E stained sections. **(C)** Quantification of renal interstitial fibrosis score in Masson’s trichrome stained sections. ^##^ *P* < 0.01, UUO group *vs.* sham group; ^*^ *P* < 0.05, **P < 0.01, UUO + HKC or UUO + losartan group *vs.* UUO group. Bar graphs represent the mean ± SEM (n = 4).

H & E staining showed that UUO mice developed typical features of obstructive nephropathy including remarkable tubular dilatation and atrophy, renal papillary necrosis, and interstitial inflammation (**Figure 1A**). HKC dose-dependently decreased kidney tubulointerstitial injury score of UUO mice (**Figure 1B**). At 1.5 g/kg, HKC ameliorated UUO-triggered tubulointerstitial injury from a score of 3.92 ± 0.21 to 2.21 ± 0.20 (*P* < 0.01, n = 4) whereas the positive control, losartan, decreased the tubulointerstitial injury from 3.92 ± 0.21 to 2.67 ± 0.24 (*P* < 0.05, n = 4) (**Figure 1B**). Masson’s trichrome staining showed a significant increase of interstitial fibrosis of UUO mice (**Figure 1A**). HKC dose-dependently decreased the interstitial fibrosis with maximal reduction of ~56% at 1.5 g/kg (**Figure 1C**). These data suggest that HKC could mitigate the renal injury in a UUO mouse model.

**Figure 2:**
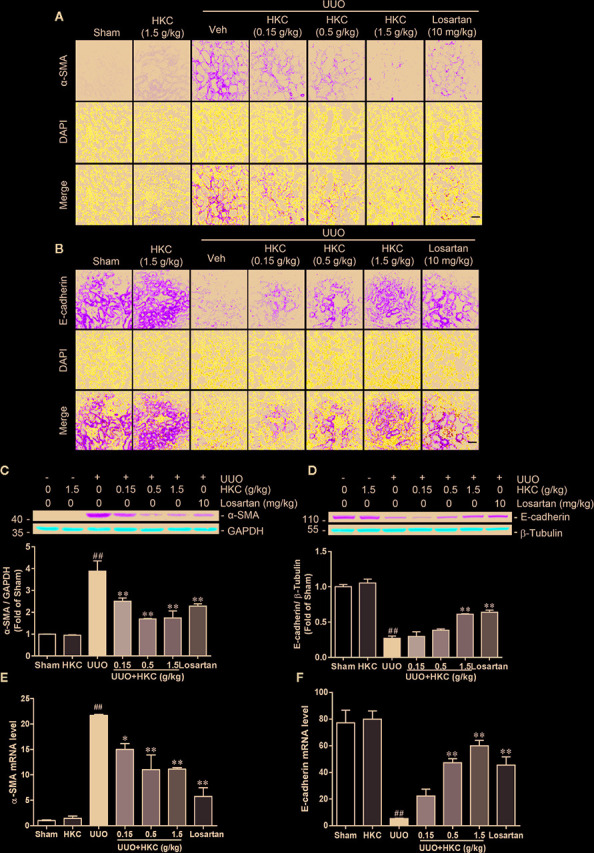
HKC suppresses α-SMA expression and increases E-cadherin expression in UUO mice. **(A)** Representative fluorescence pictures of α-SMA stained kidney slices from sham and UUO mice with different treatments. DAPI was used to stain the nuclei. Scale bar = 100 μm. **(B)** Representative fluorescence pictures of E-cadherin stained kidney slices from sham and UUO mice with different treatments. DAPI was used to stain the nuclei. Scale bar = 100 μm. **(C)** Representative western blots and quantification of α-SMA expression in kidneys from sham and UUO mice with different treatments. GAPDH was used as an internal control. **(D)** Representative western blots and quantification of E-cadherin expression in kidneys from sham and UUO mice with different treatments. β-Tubulin was used as an internal control. **(E)** mRNA levels of *α-Sma* in kidneys from sham and UUO mice with different treatments. **(F)** mRNA levels of *E-cadherin* in kidneys from sham and UUO mice with different treatments. ^##^ *P* < 0.01, UUO group *vs.* sham group; ^*^ *P* < 0.05, ^**^ *P* < 0.01, UUO + HKC group or UUO + losartan group *vs.* UUO group. Bar graphs represent the mean ± SEM (n = 3).

Given the protection of HKC on the renal fibrosis, we investigated the expression level of the fibrotic marker, α-SMA in UUO mice with or without HKC oral administration. Immunofluorescence and western blotting analysis showed that UUO mice displayed dramatic increase in the protein expression of α-SMA in the tubular interstitium compared to sham control (**Figures 2A, C** **)**. HKC dose-dependently decreased the protein expression and mRNA expression of α-SMA (**Figures 2A, C, E** **)**. Renal fibrosis is also characterized by the loss of epithelial adhesion which can be indexed by the decrease of antifibrotic protein, E-cadherin (Boutet et al., 2006). As expected, the kidneys of UUO mice displayed dramatic decrease on the protein level of E-cadherin (**Figures 2B, D** **)**. HKC dose-dependently increased the protein expression of E-cadherin to a level comparable to that of losartan (**Figures 2B, D** **)**. Consistent with the response in protein level, HKC dose-dependently increased the mRNA expression of *E-cadherin* (**Figure 2F**).

### HKC Inhibited the Expressions of Proinflammatory Mediators in UUO Mice

**Figure 3:**
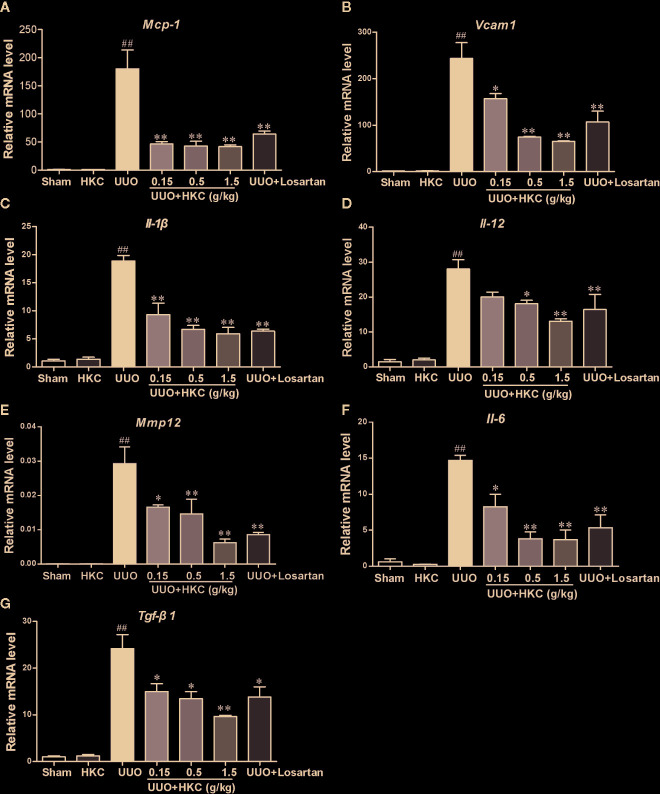
HKC suppresses the expression of inflammatory mediators in renal tissues of UUO mice. Quantitative RT-PCR analysis of MCP-1 **(A)**, VCAM1 **(B)**, IL-1β **(C)**, IL-12 **(D)**, IL-6 **(E)**, MMP12 **(F)** and TGF-β1 **(G)** levels from sham and UUO mice with different treatments. ^##^ *P* < 0.01, UUO group *vs.* sham group; ^*^ *P* < 0.05, ^**^ *P* < 0.01, UUO + HKC group *vs.* UUO group. Bar graphs represent the mean ± SEM (n = 3).

The inflammatory response is a major contributor to the progression of renal fibrosis (Meng et al., 2014). We therefore further quantitatively accessed the influence of HKC on the expression levels of a plethora of cytokines that have been reported to be involved in the renal fibrosis using qPCR analysis. UUO mice displayed higher mRNA expression levels of cytokines including interleukins (*Il-1β*, *Il-12*, *Il-6*), chemokine (*Mcp-1*), *Tgf-β*, *Mmp12*, and *Vcam1*. Administration of HKC significantly reduced mRNA levels of these cytokines in UUO mice (**Figure 3**).

### HKC Suppressed Both Canonical and Non-Canonical TGF-β Signaling Pathways

**Figure 4:**
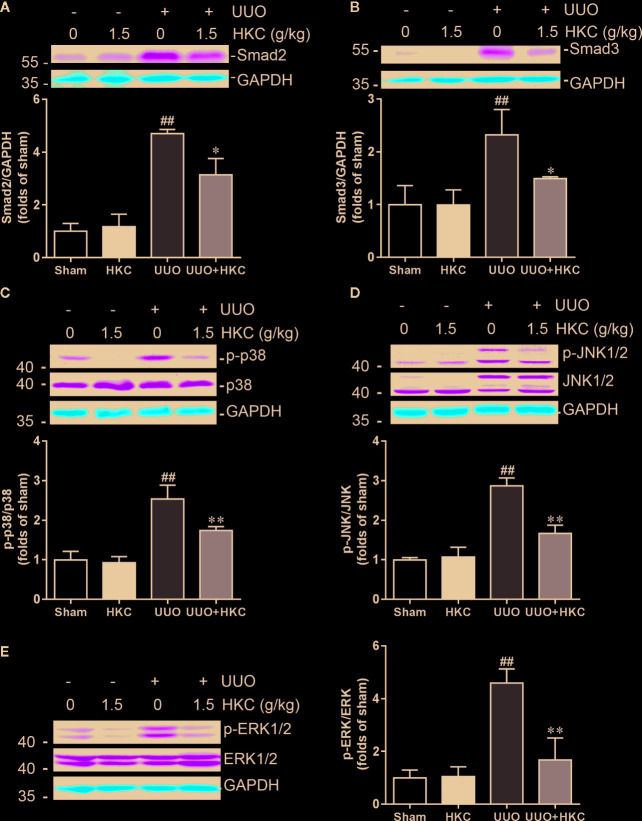
HKC treatment attenuates canonical and noncanonical TGF-β signaling pathways in UUO mice. Representative western blots and quantification of the expression levels of smad2 **(A)**, smad3 **(B)**, p-p38 **(C)**, p-JNK1/2 **(D)**, and p-ERK1/2 **(E)** in sham and UUO mice with different treatments. GAPDH was used as an internal control. ^##^ *P* < 0.01, UUO group *vs.* sham group; ^*^ *P* < 0.05, ^**^ *P* < 0.01, UUO + HKC group *vs.* UUO group. Bar graphs represent the mean ± SEM (n = 3).

TGF-β is the primary factor that drives fibrosis *via* activation of both canonical (smad2/3) and non-canonical (MAPK) signaling pathways (Meng et al., 2014). We therefore investigated whether HKC activates TGF-β dependent smad2/3 and MAPK signaling pathway. As expected, UUO mice displayed 3.71- and 1.32- fold increase of smad2 and smad3, respectively (**Figures 4A, B** **)**. HKC at 1.5 g/kg had no effect on basal expression levels of smad2/3, however, significantly decreased UUO-induced expressions of smad2 and smad3 by 42.16% and 63.88%, respectively (**Figures 4A, B** **)** demonstrating that HKC suppressed canonical TGF-β signaling pathway. UUO mice also displayed 1.54-, 1.87- and 3.60-fold increase of phosphorylated proteins of p38, JNK1/2 and ERK1/2, respectively (**Figures 4C–E**). HKC administration decreased the phosphorylated proteins of p38, JNK1/2, and ERK1/2 by 52.64, 64.21, and 81.27% respectively (**Figures 4C–E**) demonstrating that HKC also suppressed non-canonical TGF-β signaling pathway.

Deletion of *Trpc6* Decreased smad2/3 Expression and MAPK Phosphorylation in UUO Mice

**Figure 5:**
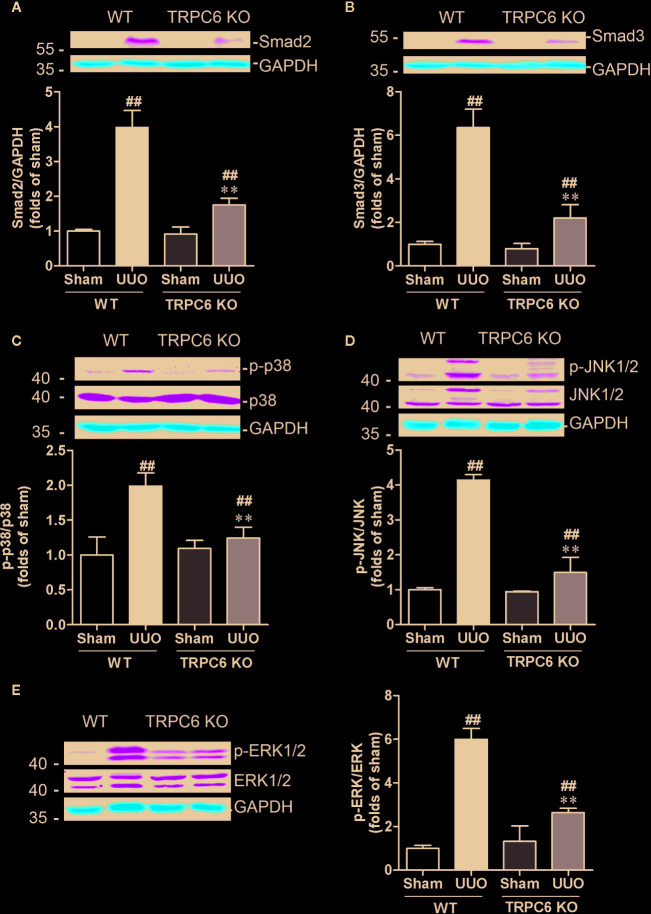
Deletion of *Trpc6* inhibits canonical and non-canonical TGF-β signaling pathways in UUO mice. Representative western blots and quantification of the levels of smad2 **(A)**, smad3 **(B)** and phosphorylation levels of p38 **(C)**, JNK1/2 **(D)**, and ERK1/2 **(E)** in WT and TRPC6 KO mice. GAPDH was used as an internal control. ^##^ *P* < 0.01, UUO group *vs.* sham group in each genotype; ^**^ *P* < 0.01, TRPC6 KO *vs.* WT in UUO mice. Bar graphs represent the mean ± SEM (n = 3).

Recent studies have shown that TRPC6 is a pivotal player in the progression of renal fibrosis. In UUO mice, the expression level of TRPC6 is increased and genetic ablation or pharmacological inhibition of TRPC6 suppresses UUO-induced interstitial fibrosis (Wu et al., 2017). We therefore first investigated whether deletion of *Trpc6* suppressed the TGF-β signaling pathway that mimics the effect of HKC. TRPC6 KO mice displayed significantly decreased UUO-induced protein expressions of smad2 and smad3 by 74.77% and 77.50%, respectively (**Figures 5A, B** **)**. Deletion of TRPC6 also significantly decreased UUO-induced phosphorylation levels of p38, JNK1/2 and ERK1/2 by 75.53, 84.22, and 67.22%, respectively (**Figures 5C–E**) suggesting that TRPC6 also regulates both canonical and non-canonical TGF-β signaling pathways.

### HKC Suppressed TRPC6 Expression in the Kidney of UUO Mouse

**Figure 6:**
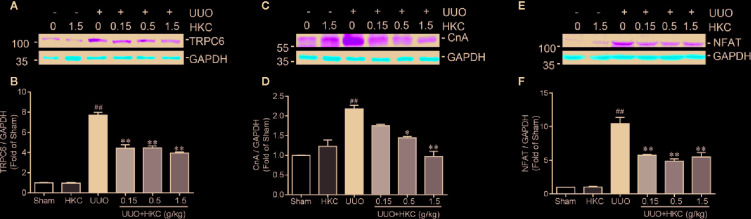
HKC suppresses TRPC6/CnA/NFAT expression in UUO mice. Representative western blots and quantification of the levels of TRPC6 **(A)**, CnA **(B)** as well as NFAT **(C)** in sham, and UUO mice with different treatments. GAPDH was used as an internal control. ^##^ *P* < 0.01, UUO group *vs.* sham group; ^*^ *P* < 0.05, ^**^ *P* < 0.01, UUO + HKC group *vs.* UUO group. Bar graphs represent the mean ± SEM (n = 3).

Given the inhibitory effect on TGF-β signaling of HKC treatment and deletion of *Trpc6*, we therefore investigated HKC effect on TRPC6 expression in UUO mice. Consistent with previous demonstration (Wu et al., 2017), UUO mice displayed increased protein expression of TRPC6 (**Figures 6A, B** **)**. HKC had no effect on basal level of TRPC6 expression. However, administration of HKC suppressed the TRPC6 expression in the kidneys of UUO mice (**Figures 6A, B** **)**. TRPC6 mediated Ca^2+^ influx induces CnA/NFAT activation that is necessary in the progression of pulmonary fibrosis and myofibroblast transformation (Davis et al., 2012; Hofmann et al., 2017). UUO mice displayed higher expression levels of CnA and NFAT that were inhibited by HKC administration (**Figures 6C–F**).

### HKC Directly Suppressed TRPC3/6 Activities in Heterologous Expression System

**Figure 7:**
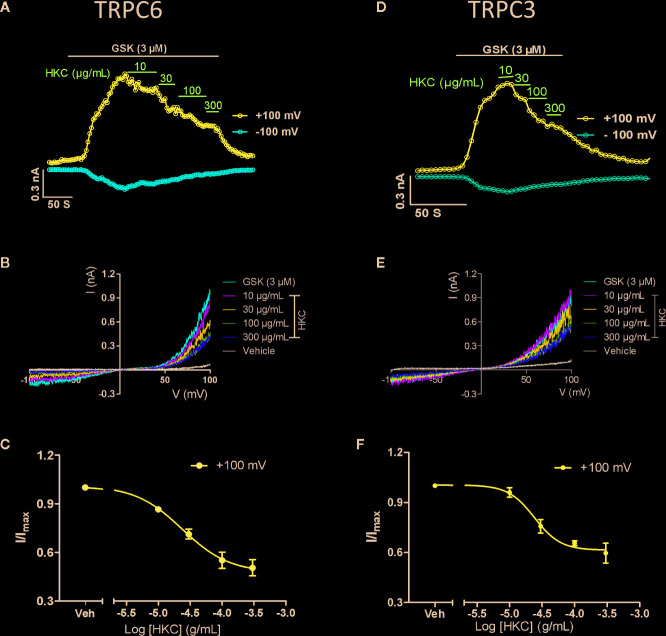
Inhibitory effect of HKC on TRPC3/6 activity in HEK-293 cells expressing TRPC3 or TRPC6 channels. **(A)** Representative traces of whole-cell currents recorded at +100 (blue traces) and −100 mV (red traces) induced by GSK1702934A (3 μM) in HEK-293 cells expressing TRPC6. **(B)** Current-voltage (I-V) curves induced by GSK1702934A in the presence of vehicle (Veh, 0.1% DMSO) or different concentration of HKC. **(C)** Concentration-response curves for HKC inhibition of TRPC6 current recorded at +100 mV. **(D)** Representative traces of whole-cell currents recorded at +100 (blue traces) and −100 mV (red traces) induced by GSK1702934A (3 μM) in HEK-293 cells expressing TRPC3. **(E)** Current-voltage (I-V) curves induced by GSK1702934A in the presence of vehicle (Veh, 0.1% DMSO) or different concentration of HKC. **(F)** Concentration-response curves for HKC inhibition of TRPC3 current recorded at +100 mV. Each data point represents the mean ± SEM (n = 4).

Research has proposed that TRPC6 and NFAT form mutually positive feedback loop that exacerbates the renal fibrosis (Nijenhuis et al., 2011). We therefore investigated whether HKC, as a whole, was able to directly suppress TRPC6 activity in heterologous expression system. GSK1702934A is a potent TRPC3/6 agonist (Qu et al., 2017; Yang et al., 2019). In HEK-293 cells expressing TRPC6, bath application of GSK1702934A (3 μM) induced a doubly rectified current with a reversal potential close to 0 mV (**Figures 7A, B** **)**. Co-application of HKC (10–300 µg/mL) concentration-dependently suppressed the outward (+100 mV) currents with IC_50_ values of 23.70 μg/mL (**Figures 7A–C**). TRPC3 is highly homologous to TRPC6 and TRPC3 KO mouse also displayed attenuated renal fibrosis in a UUO mouse model (Wu et al., 2017). We therefore also tested the effect of HKC on GSK1702934A-induced currents in HEK-293 cells expressing TRPC3. Similar to TRPC6, we observed a concentration-dependent inhibition of HKC on outward (+100 mV) current of TRPC3 with IC_50_ values of 24.07 µg/mL (**Figures 7D–F**).

### HKC Inhibited UUO Renal Fibrosis Through TRPC6 Channels

**Figure 8:**
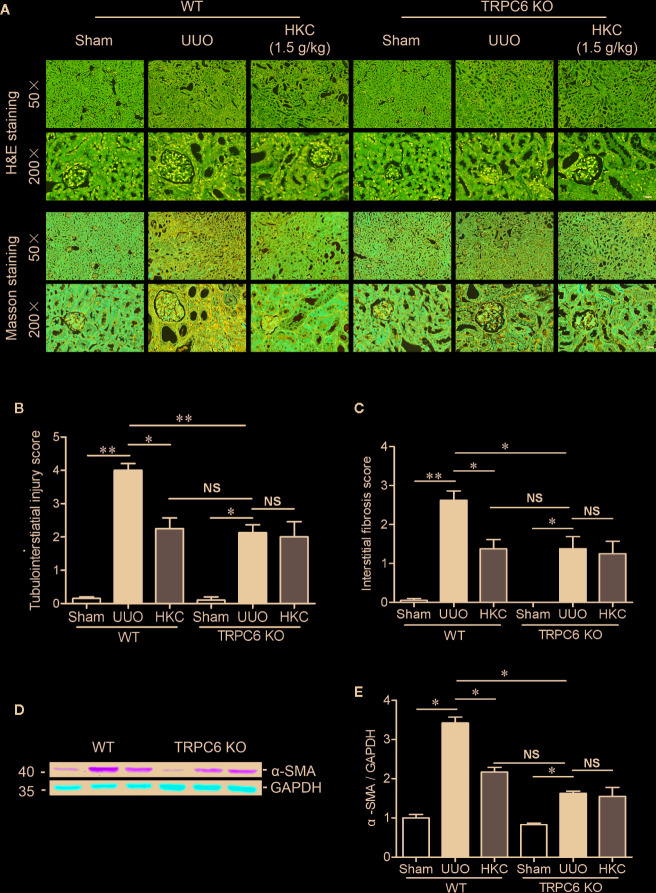
HKC treated wild type mice and TRPC6 knock out (KO) mice display comparable, but not additive protection, on the tubulointerstiatial injury and interstitial fibrosis triggered by UUO. **(A)** Representative images of H & E (upper two panels) and Masson’s trichrome staining (lower two panels) of the kidneys from WT and TRPC6 KO mice subjected to sham or UUO with or without HKC (1.5 g/kg) treatment. Scale bar = 500 μm. **(B)** Quantitation of tubulointerstiatial injury score derived from H & E staining. **(C)** Quantitation of renal interstitial fibrosis score derived from Masson’s trichrome staining. **(D)** Representative western blots of α-SMA expression in the kidneys from WT and TRPC6 KO mice subjected to sham or UUO with or without HKC treatment. GAPDH was used as an internal control. **(E)** Quantification of the levels of α-SMA. ^*^ *P* < 0.05, ^**^ *P* < 0.01. *^NS^ no statistical significance.* Bar graph represents the mean ± SEM (n = 3).

Given the direct inhibition of TRPC6 activity of HKC, we next compared the HKC protective effect in both WT and TRPC6 KO mice that were subjected to UUO surgery. In TRPC6 KO mice, tubular damage as well as extracellular matrix deposition was alleviated to a level comparable to that observed in WT UUO mice with HKC administration at a dose of 1.5 g/kg/day (**Figures 8A–C**). Administration of HKC in TRPC6 KO mice with UUO surgery didn’t display any protection on the renal damage (**Figures 8A, B** **)**. Similarly, the degree of HKC (1.5 g/kg/day) suppressed renal fibrosis in WT UUO mice is comparable to that observed in TRPC6 KO mice after UUO (**Figures 8A, C** **)**. HKC administration didn’t suppress UUO-induced fibrosis in TRPC6 KO mice with UUO surgery (**Figures 8A** **, C**). In WT mice, the α-SMA level was 3.42-fold higher than sham control. Deletion of *Trpc6* decreased α-SMA expression by 74.12%, a reduction comparable by HKC treatment in WT UUO mice. Administration of HKC didn’t decrease the α-SMA level in TRPC6 KO mice with UUO surgery (**Figures 8D, E** **)**.

## Discussion

Renal fibrosis is the final common pathological manifestation of CKD (Boor et al., 2010; Eddy, 2014). Increased expression of α-SMA and decreased expression of E-cadherin are considered to be the major hallmarks of tubular epithelial-mesenchymal transition (EMT) that lead to the destruction of renal parenchyma (Liu, 2011). Consistent with previous study (Gu et al., 2018), we demonstrated that UUO mice displayed increased expression of α-SMA and decreased expression of E-cadherin suggesting the loss of epithelial and gain of mesenchymal features. HKC treatment significantly decreased the expression of α-SMA and increased the expression of E-cadherin. Consistent with biochemical investigation, HKC also ameliorates the UUO-induced kidney tubulointerstitial injury and interstitial fibrosis.

Although inflammation is an evolutionary defense mechanism following injury and infectious agents, excessive inflammatory cells infiltration promotes the progression of renal fibrosis (Nathan and Ding, 2010; Liu, 2011). We demonstrated that HKC significantly suppressed the expression levels of MCP-1 (chemokine produced by tubular cells) (Vieira et al., 2005), VCAM-1 (monocyte marker) (Chuang et al., 2015), inflammation mediators produced by M1 macrophages such as IL-1β, IL-12, IL-6 and MMP-12 (Shen et al., 2014) and TGF-β, an indicator of M2 macrophages (Meng et al., 2014; Shen et al., 2014). Considered together, these data demonstrated that HKC suppressed the recruitments of monocytes/macrophages that drive the progression of renal fibrosis.

TGF-β, one of the central fibrotic factors, plays a major role in the trans-differentiation of interstitial fibroblast to myofibroblast during renal fibrosis by activation both canonic and non-canonic signaling pathways which involve smad2/3 and MAPK proteins, respectively (Geng et al., 2019). We demonstrated that HKC suppressed both smad2/3 expression and phosphorylated protein of p38, JNK1/2, and ERK1/2 suggesting that HKC was capable of suppressing both canonic and non-canonic TGF-β signaling pathways. This is consistent with the report that HKC attenuates renal inflammation and glomerular injury through inhibition of ROS-MAPK signaling pathway (Tu et al., 2013; Mao et al., 2015; Li et al., 2019).

An interesting finding is that HKC protection against renal fibrosis is through TRPC6 channels. TRPC6 is a Ca^2+^ permeable, non-selective cation channels that have been shown to be involved in renal fibrosis (Dryer et al., 2019; Lin et al., 2019). Consistent with previous finding, we demonstrated that UUO stimulated TRPC6 expression and the downstream proteins CnA/NFAT that are necessary in the programming myofibroblast transformation (Davis et al., 2012; Hofmann et al., 2017). Overexpression of TRPC6 has been shown to activate myofibroblast transformation, while fibroblasts lacking TRPC6 were insensitive to TGF-β and angiotensin II-induced trans-differentiation (Davis et al., 2012). HKC significantly suppressed UUO-induced expressions of TRPC6, CnA, and NFAT suggesting that HKC protection against renal fibrosis is likely through TRPC6/CnA/NFAT signaling pathway. In TRPC6 KO mice, we observed reductions on both the protein expression of smad2/3 and the phosphorylated proteins of MAPK demonstrating that genetic ablation of TRPC6 attenuated both canonic and non-canonic TGF-β signaling pathways, a phenomenon consistent with HKC inhibition on TGF-β signaling pathways. The strongest evidence is that TRPC6 KO mice and HKC treated WT mice displayed comparable reductions on the tubulointerstitial injury score, interstitial fibrosis score, and α-SMA expression. Furthermore, administration of HKC in TRPC6 KO mice subjected to UUO didn’t display any protection. Considered together, these data demonstrate that HKC protection against renal fibrosis is through TRPC6 dependent pathway. It should be noted that the expression levels of smad2 and smad3 in HKC-treated are higher than that observed in TRPC6 KO mice subjected to UUO. Such difference is likely due to the fact that HKC decreased UUO-induced TRPC6 expression is not complete (56% reduction) while TRPC6 KO is completely absence of TRPC6 expression.

Research has demonstrated that TRPC6 and NFAT form mutually positive feedback loop that exacerbates the renal fibrosis (Nijenhuis et al., 2011). Therefore, it is likely that direct suppression of TRPC6 activity contributes to HKC inhibited TRPC6/CnA/NFAT expression, which is responsible for TGF-β signaling critical for the progression of renal fibrosis. We demonstrate that TRPC6 is a direct target of HKC suggesting that suppression of TRPC6 activity by HKC may contributes to its renal protection. However, whether the direct inhibition of TRPC6 is exclusively responsible for HKC protection against renal fibrosis is arguable since flavonoids, the main active compounds in HKC, have been reported to modulate inflammation (Lu et al., 2018), a critical factor driving renal fibrosis. In addition, we also observed a direct inhibition of TRPC3 by HKC, a structural highly homologous protein to TRPC6 (Tang et al., 2018). Genetic ablation of TRPC3 has been shown to attenuate renal fibrosis in UUO mouse model. However, double TRPC3/6 KO confers the same degree of protection against kidney fibrosis as single TRPC6 deletion (Wu et al., 2017) suggesting that TRPC3 and TRPC6 work in the same pathway, possibly by formation of TRPC3/TRPC6 heteromultimers.

In summary, we demonstrate that HKC, a clinically used anti-proteinuria drug, attenuates tubulointerstitial injury and kidney interstitial fibrosis in a UUO mouse model. We further demonstrate that HKC protection of renal fibrosis is dependent on TRPC6 pathway, possibly through direct inhibition of TRPC6 channel activity and indirect suppression of TRPC6 expression.

## Data Availability Statement

All datasets presented in this study are included in the article/**Supplementary Material**.

## Ethics Statement

All the animal care and experimental protocols were approved by the Animal Ethics Committee of China Pharmaceutical University according to Guidelines of the National Institutes of Health for the Care and Use of Laboratory Animals.

## Author Contributions

All authors contributed to the article and approved the submitted version. L-FG, LZ, H-TG, and Y-JW performed the experiments and analyzed data. L-FG drafted the manuscript. FZ, Z-YC and H-TT participated in data analysis. B-YY, Z-YC, and C-ZC designed the experiments and proofed the paper.

## Funding

This work was supported by the National Natural Science Foundation of China (81972960, 21777192) and “Double First-Class” University project (CPU2018GF06).

## Conflict of Interest

Authors H-TG and HT-T are employed by the Suzhong Pharmaceutical Group Co., Ltd.

The remaining authors declare that the research was conducted in the absence of any commercial or financial relationships that could be construed as a potential conflict of interest. 
