# Supplementary figures and images for: Huangkui Capsule Ameliorates Renal Fibrosis in a Unilateral Ureteral Obstruction Mouse Model Through TRPC6 Dependent Signaling Pathways
Source: Front Pharmacol. 2020 Jul 3;11:996. doi: 10.3389/fphar.2020.00996 (PMC7350529; doi:10.3389/fphar.2020.00996)

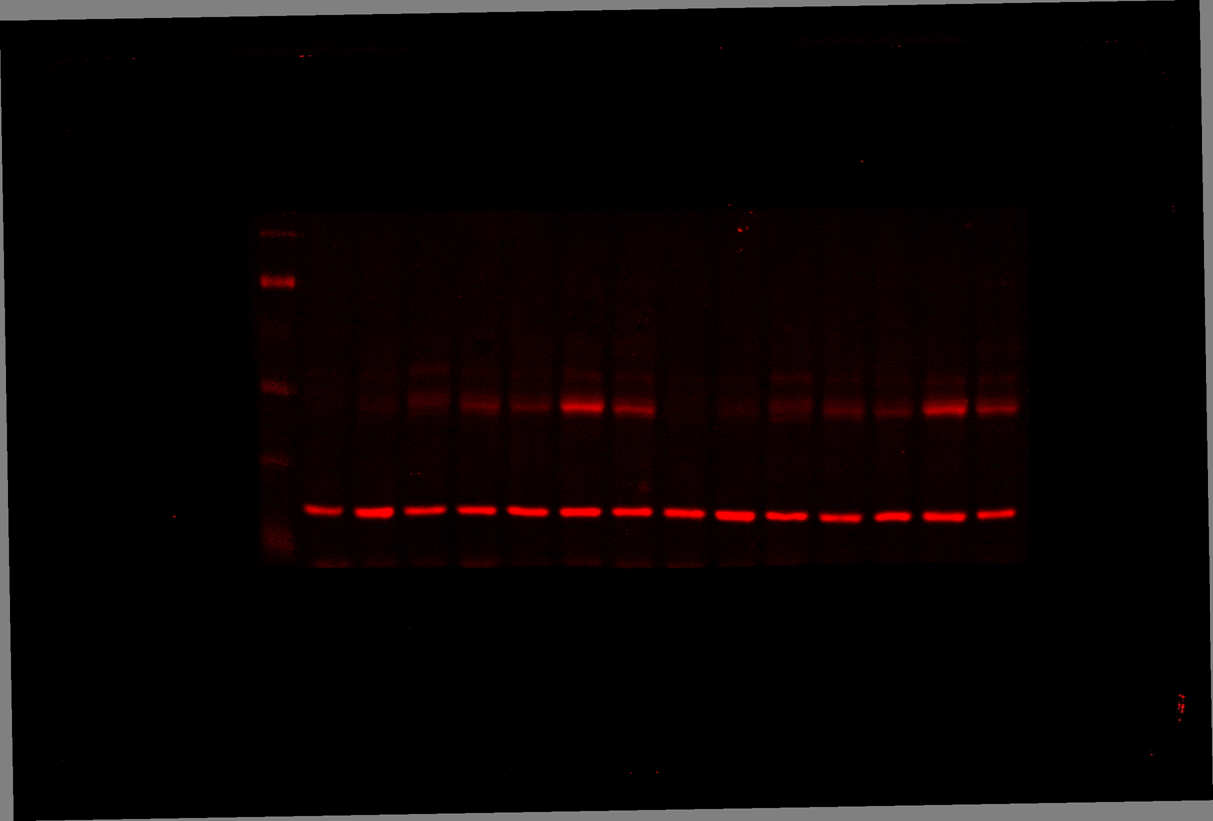

Supplement: Supplementary file 1 [file DataSheet_1.zip › WB/10-CnA-GA.tif]

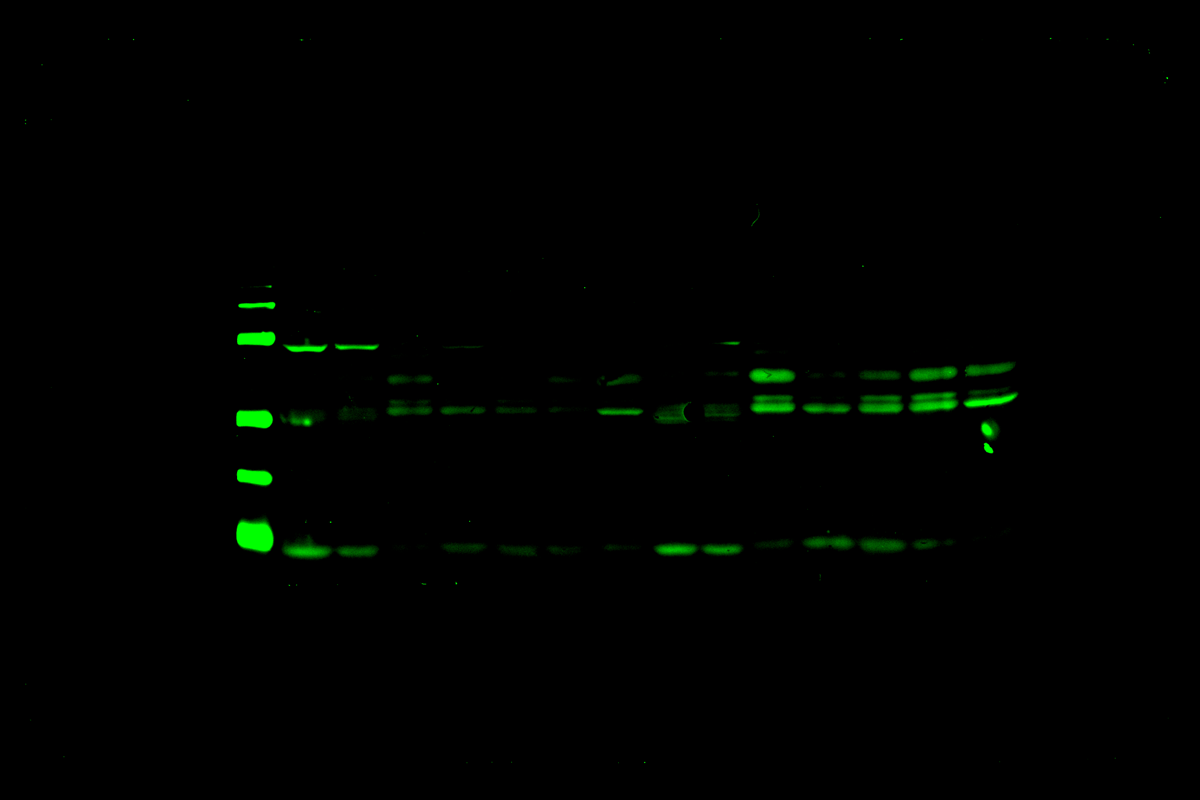

Supplement: Supplementary file 1 [file DataSheet_1.zip › WB/11-CnA2.tif]

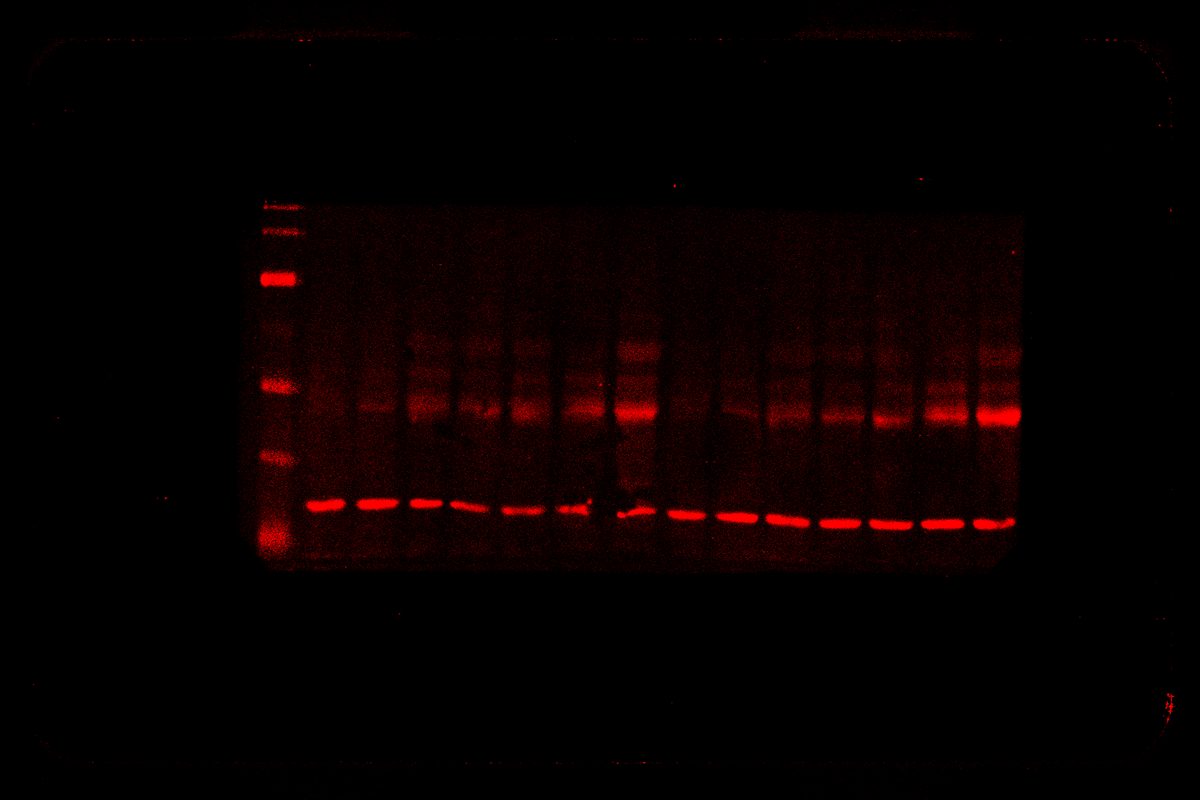

Supplement: Supplementary file 1 [file DataSheet_1.zip › WB/12-CnA-GA2.tif]

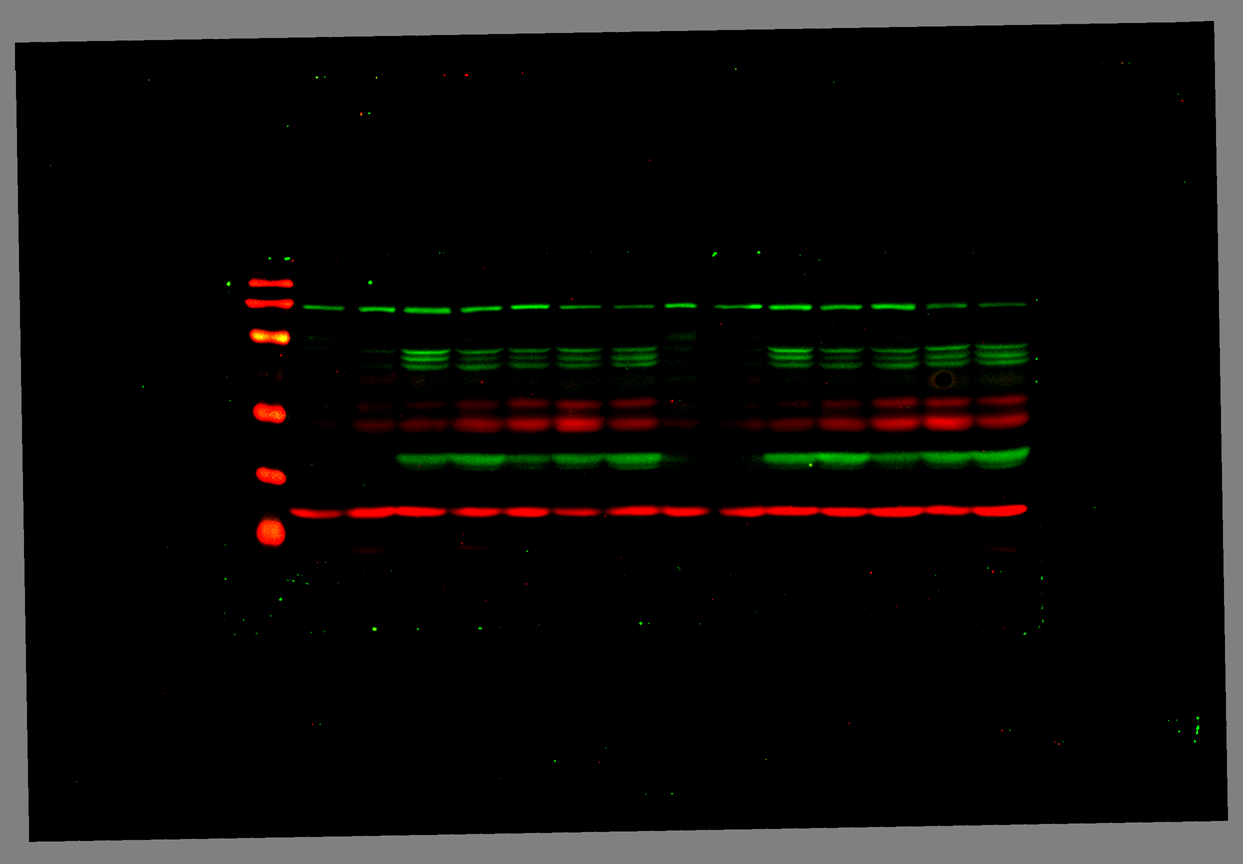

Supplement: Supplementary file 1 [file DataSheet_1.zip › WB/13-NFAT.tif]

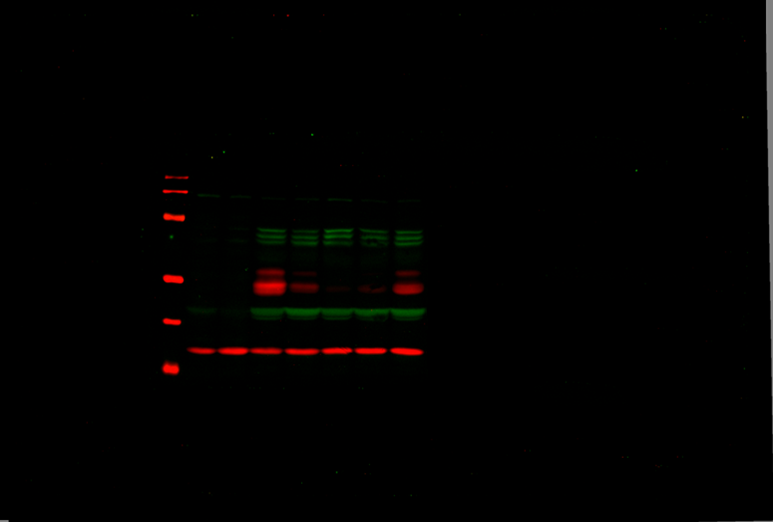

Supplement: Supplementary file 1 [file DataSheet_1.zip › WB/14-NFAT-2.tif]

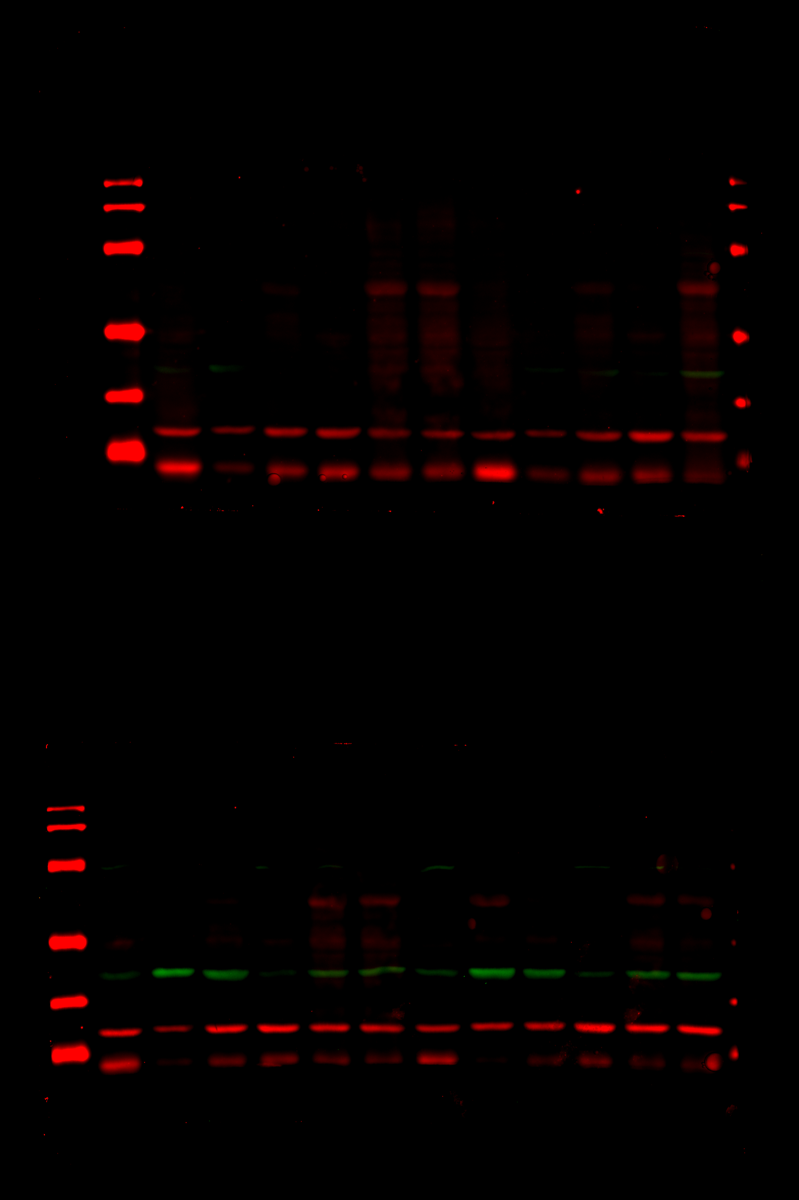

Supplement: Supplementary file 1 [file DataSheet_1.zip › WB/15-SMA-KO-down.tif]

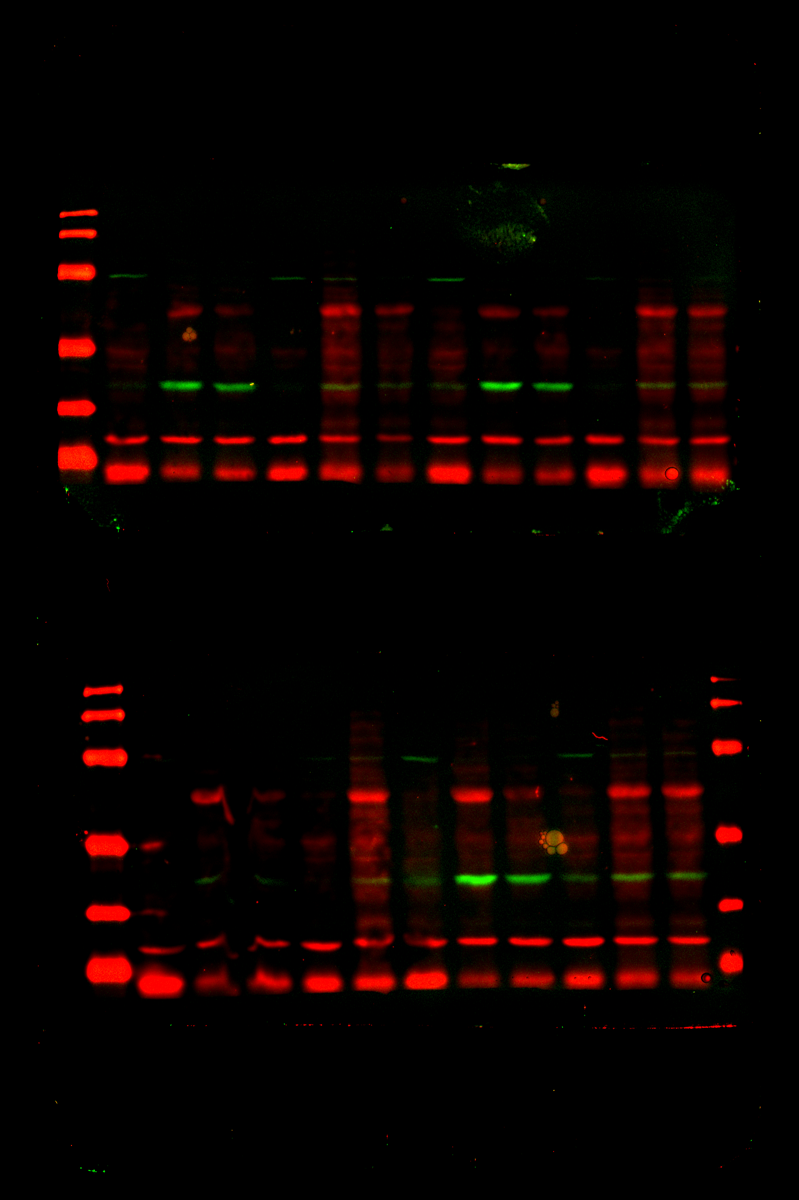

Supplement: Supplementary file 1 [file DataSheet_1.zip › WB/16-SMA-KO-2-up.tif]

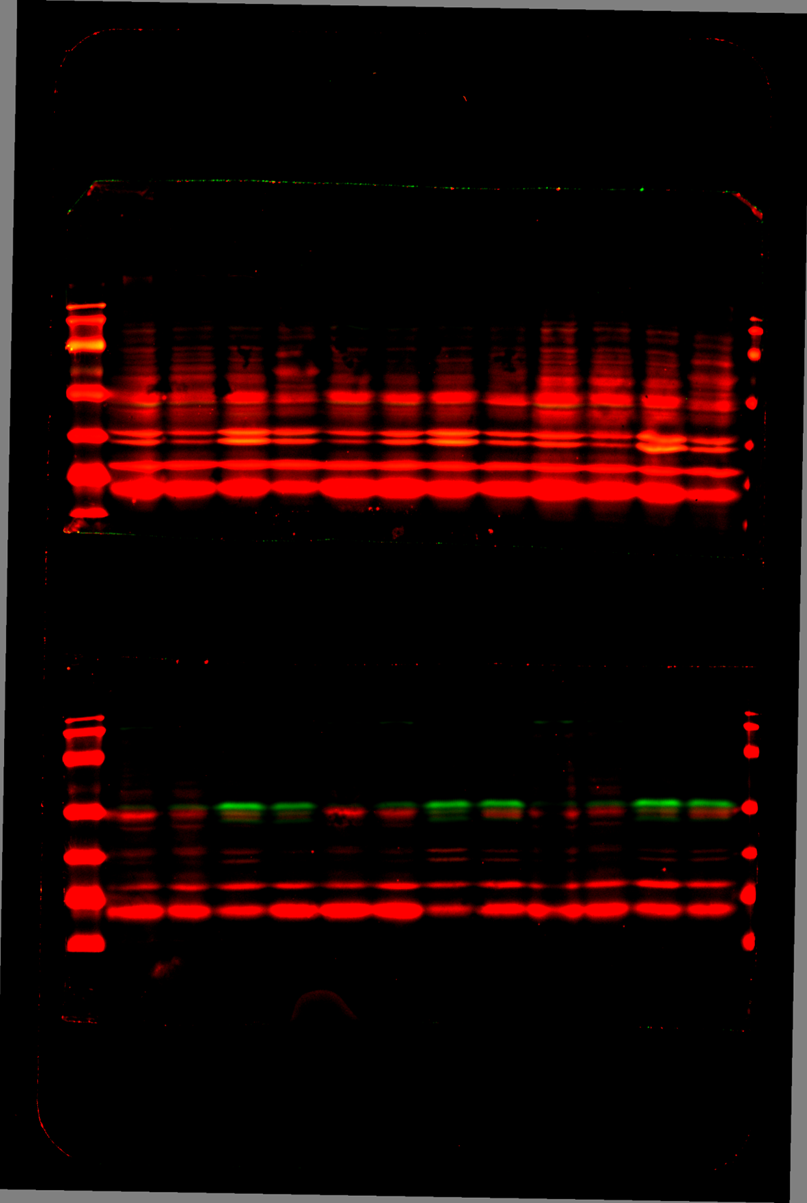

Supplement: Supplementary file 1 [file DataSheet_1.zip › WB/17-Smad2-HKC-down.tif]

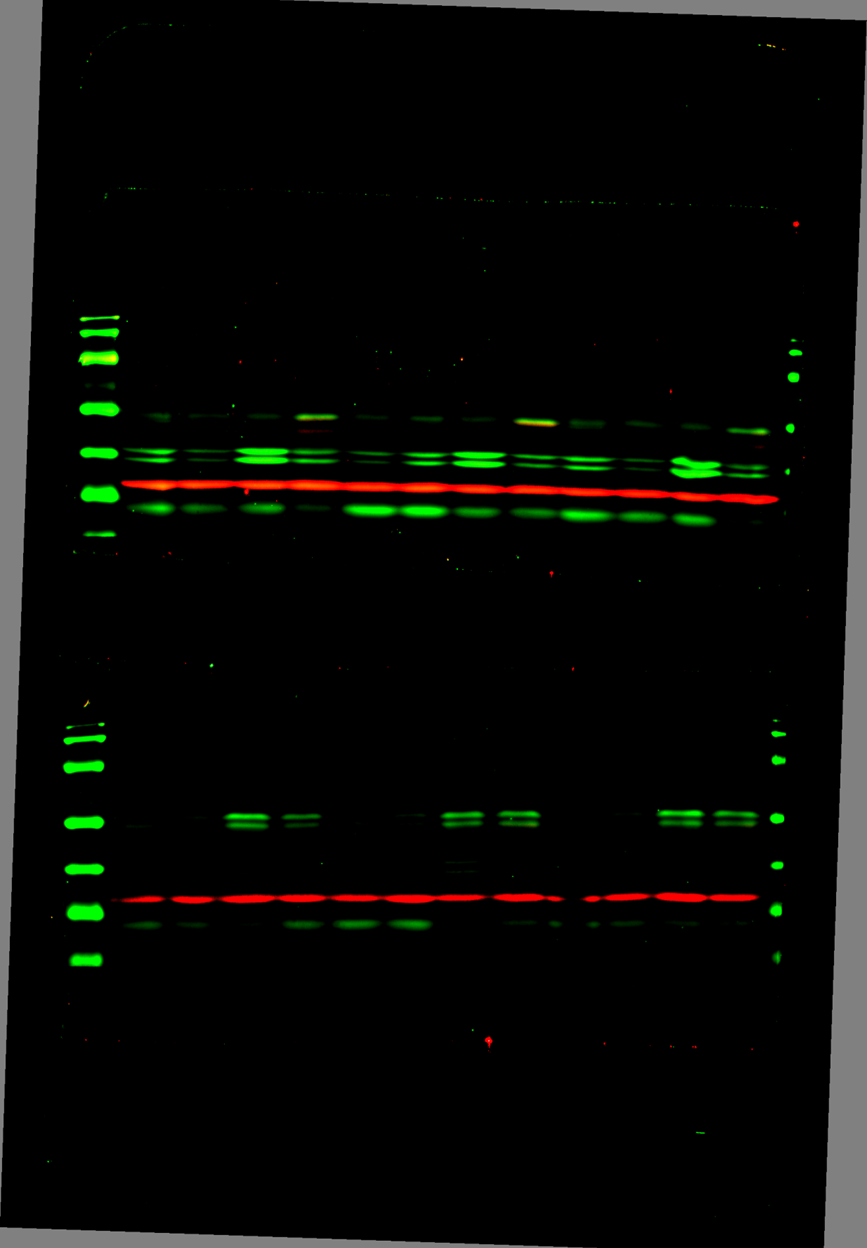

Supplement: Supplementary file 1 [file DataSheet_1.zip › WB/18-Smad3-HKC-down.tif]

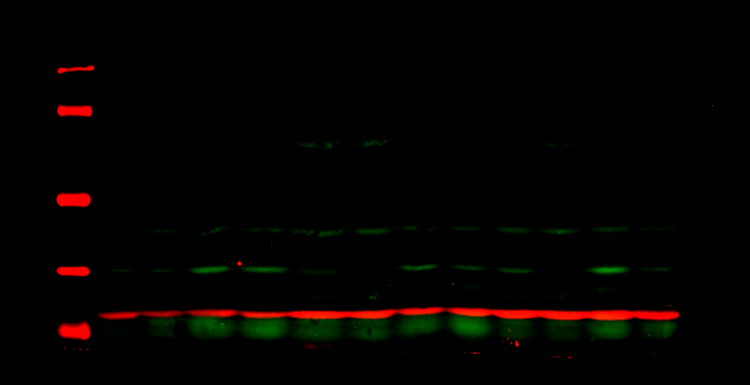

Supplement: Supplementary file 1 [file DataSheet_1.zip › WB/19-pp38-HKC.tif]

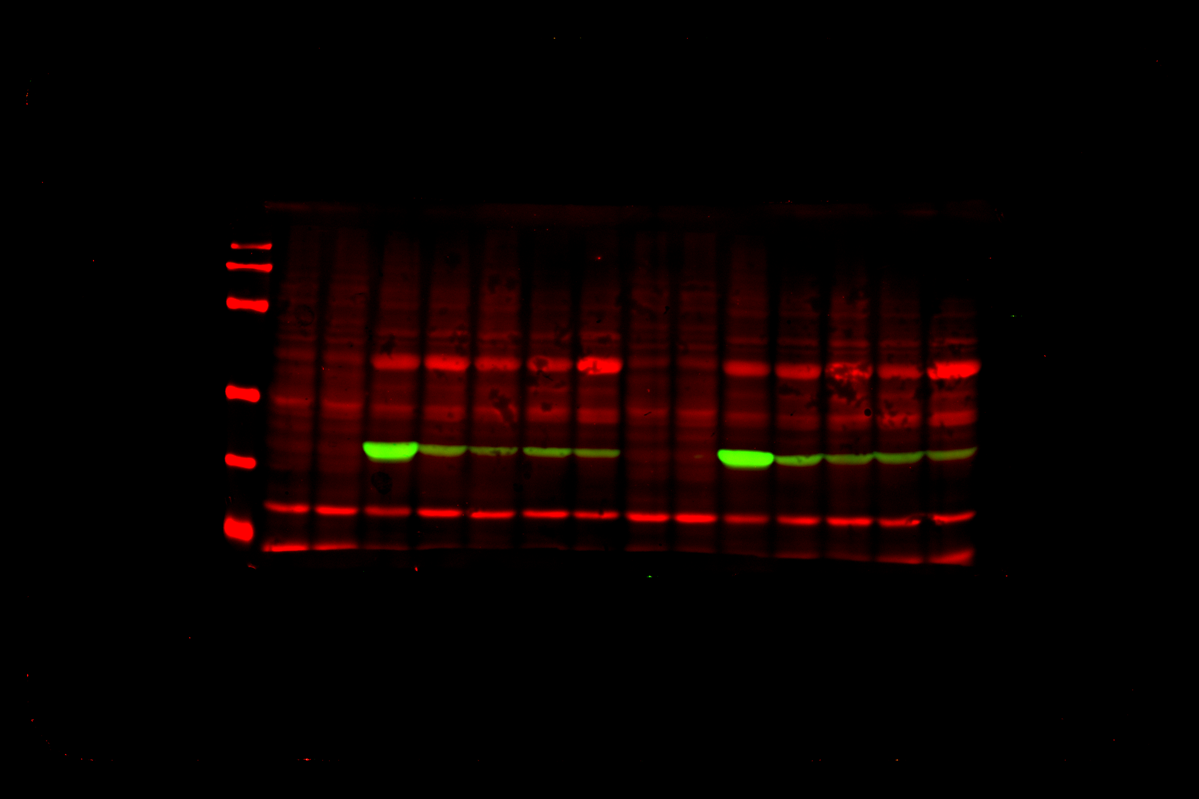

Supplement: Supplementary file 1 [file DataSheet_1.zip › WB/1-SMA.tif]

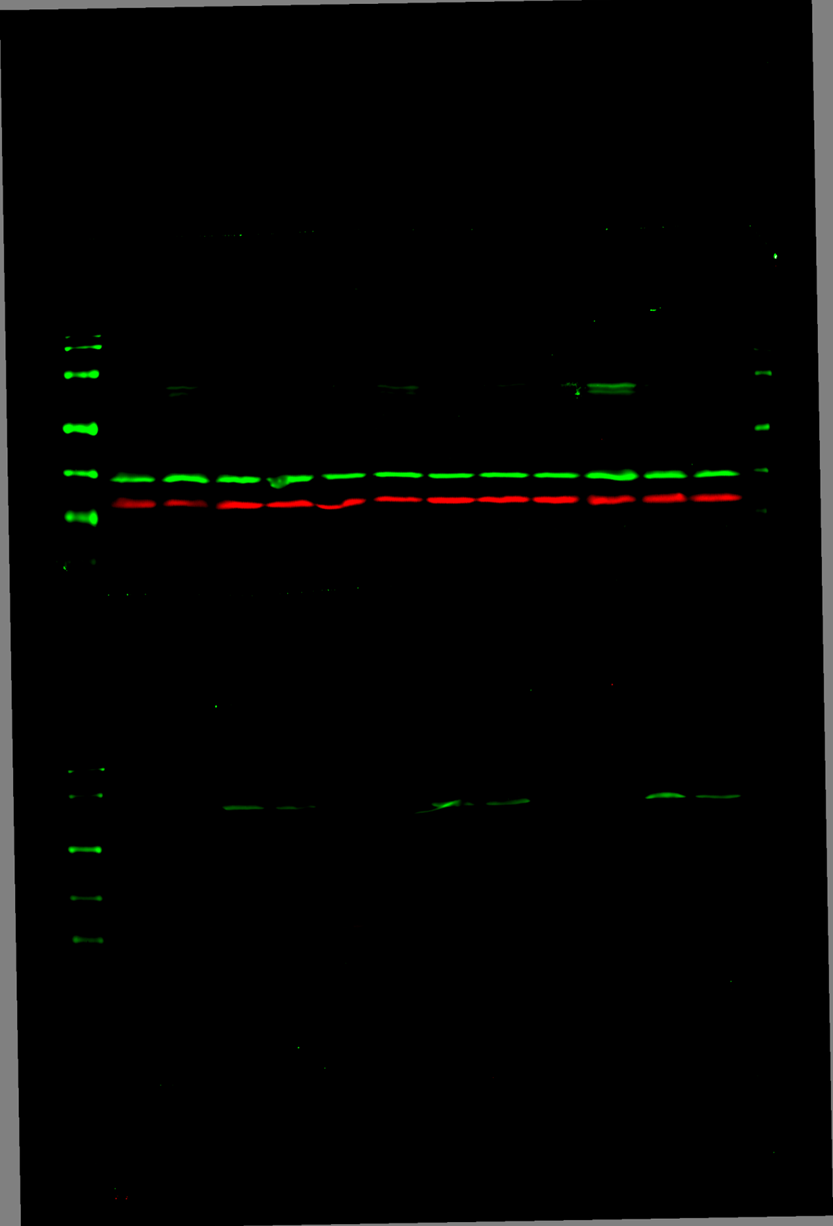

Supplement: Supplementary file 1 [file DataSheet_1.zip › WB/20-p38-HKC-up.tif]

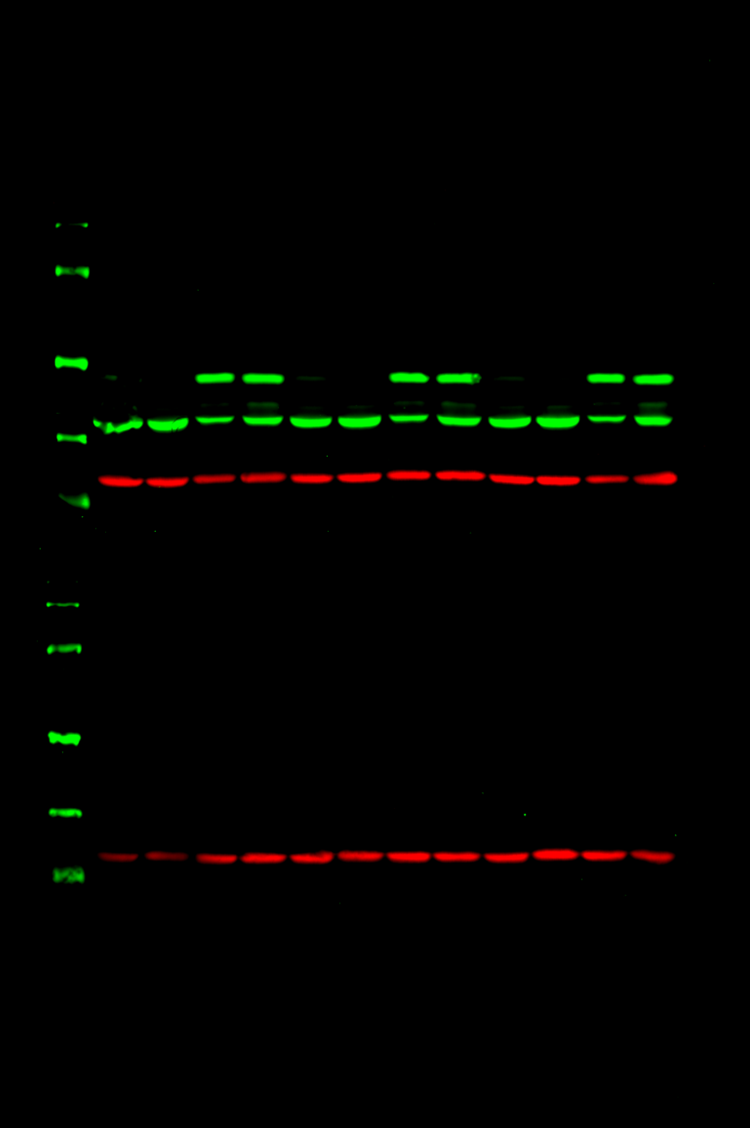

Supplement: Supplementary file 1 [file DataSheet_1.zip › WB/21-JNK-HKC-up.tif]

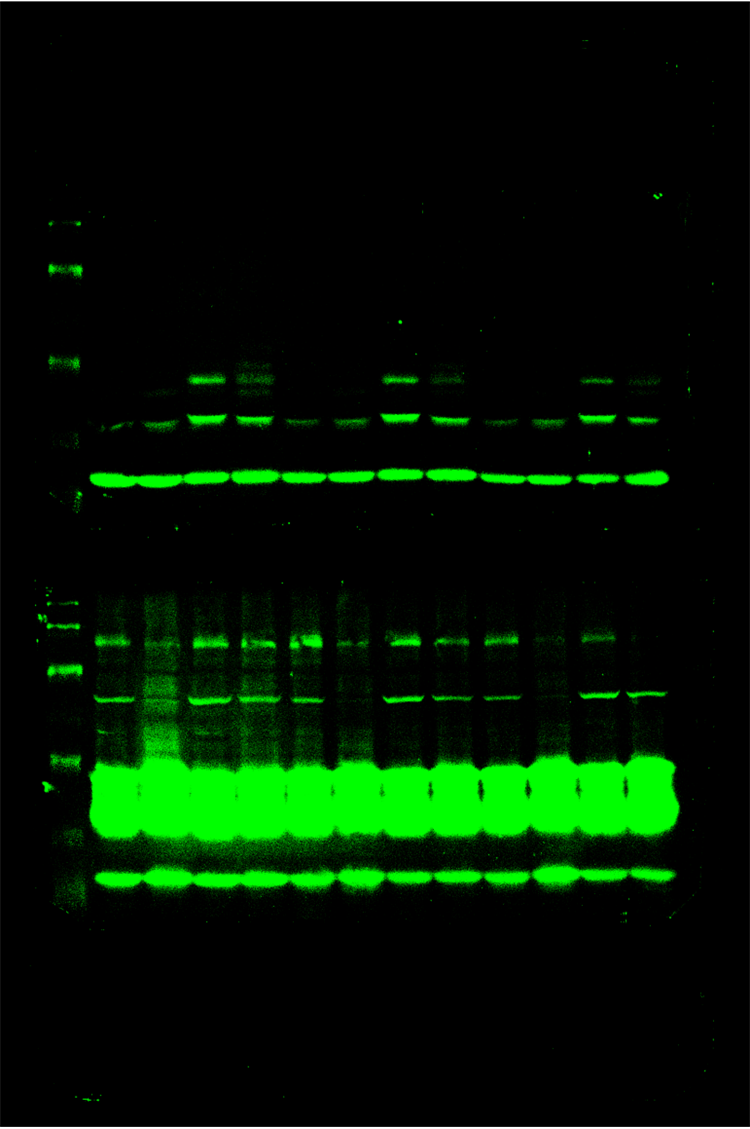

Supplement: Supplementary file 1 [file DataSheet_1.zip › WB/22-pJNK-HKC-up.tif]

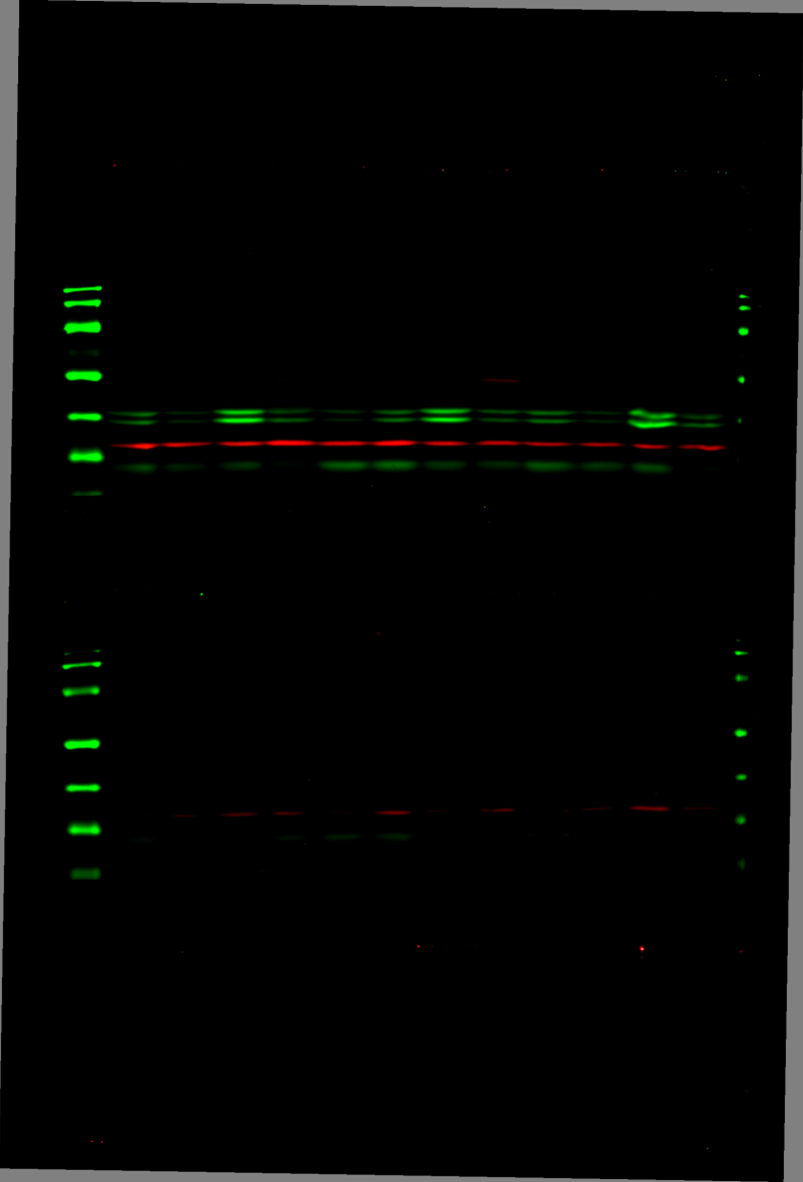

Supplement: Supplementary file 1 [file DataSheet_1.zip › WB/23-pERK-HKC-up.tif]

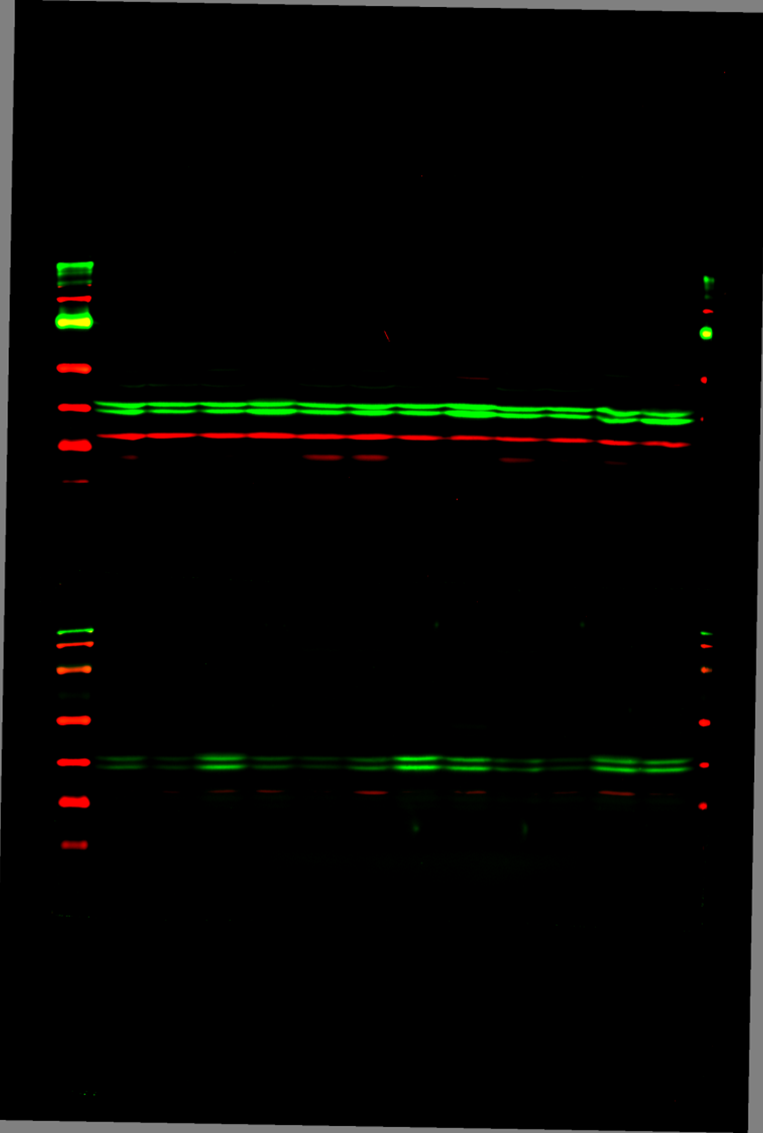

Supplement: Supplementary file 1 [file DataSheet_1.zip › WB/24-ERK-HKC-up.tif]

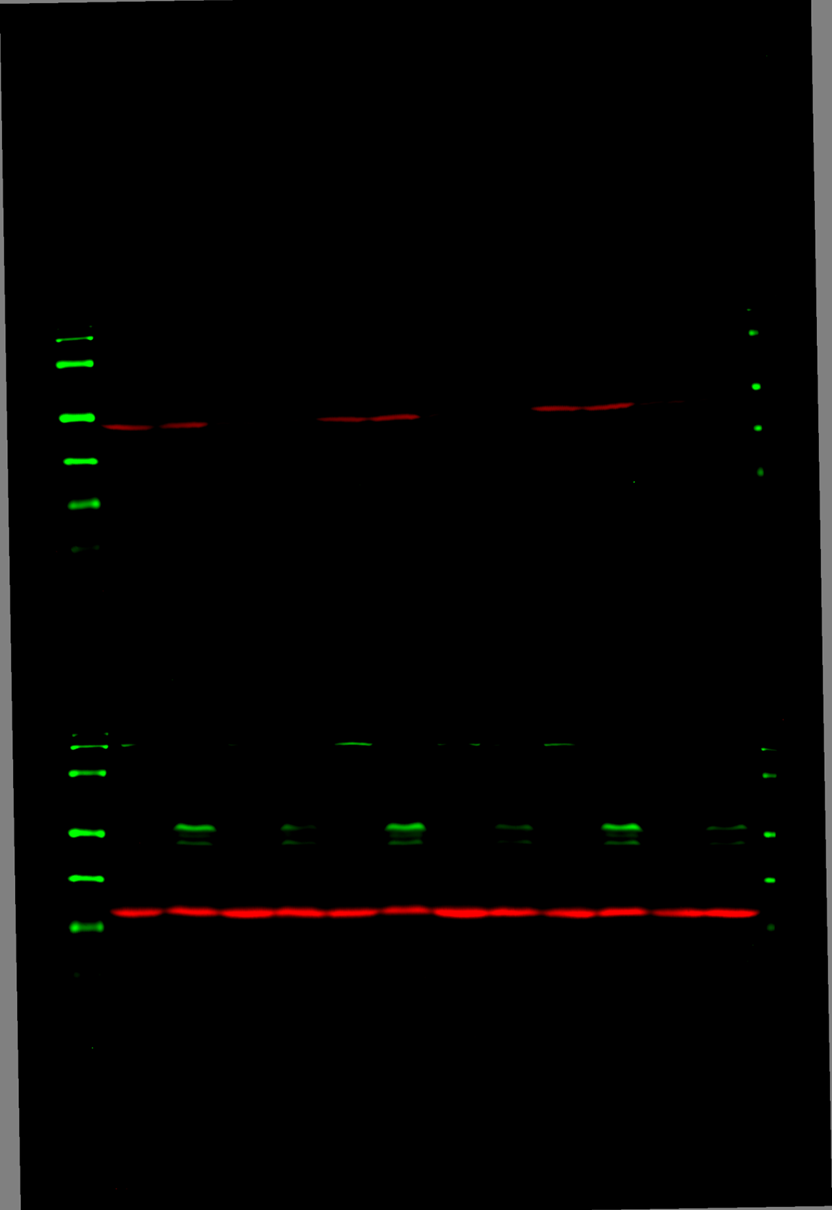

Supplement: Supplementary file 1 [file DataSheet_1.zip › WB/25-Smad2-KO-down.tif]

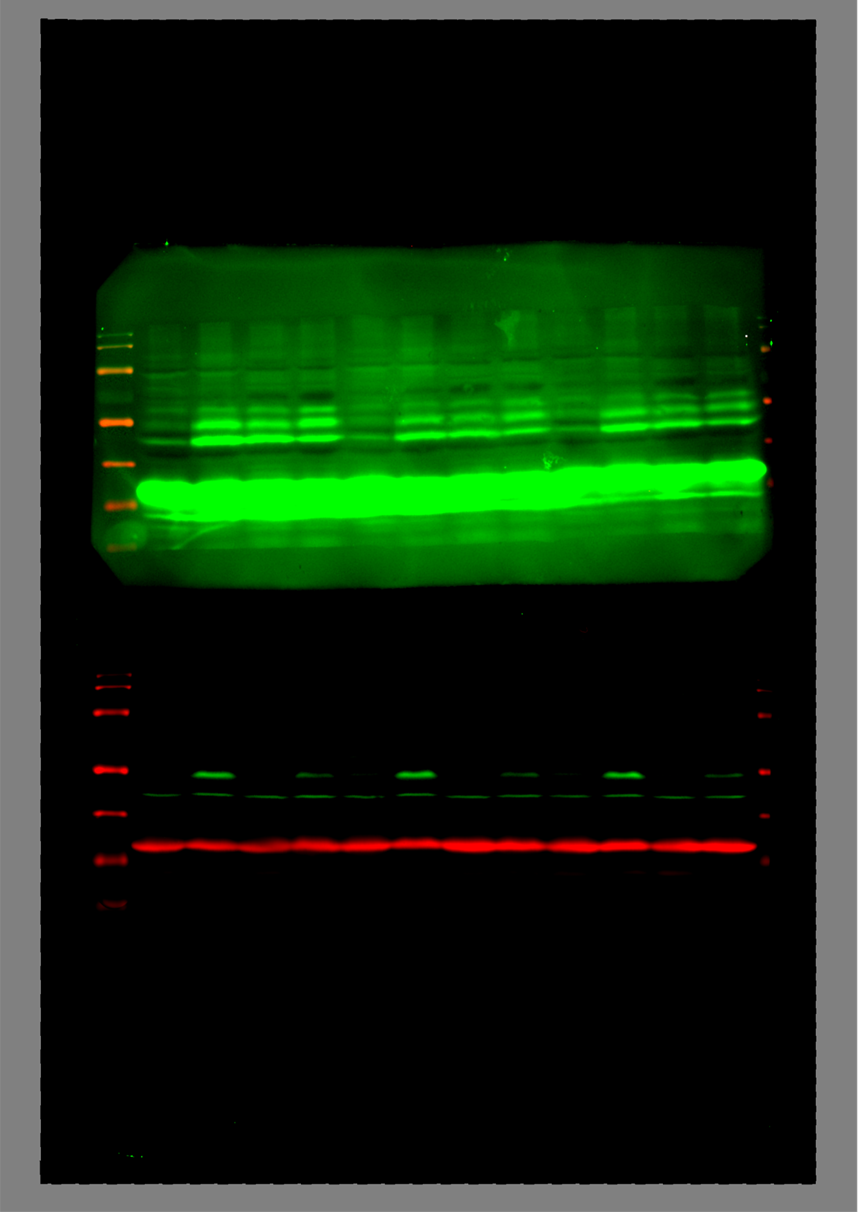

Supplement: Supplementary file 1 [file DataSheet_1.zip › WB/26-SMA3-KO-down.tif]

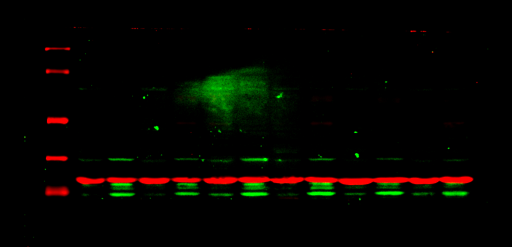

Supplement: Supplementary file 1 [file DataSheet_1.zip › WB/27-pp38-KO.tif]

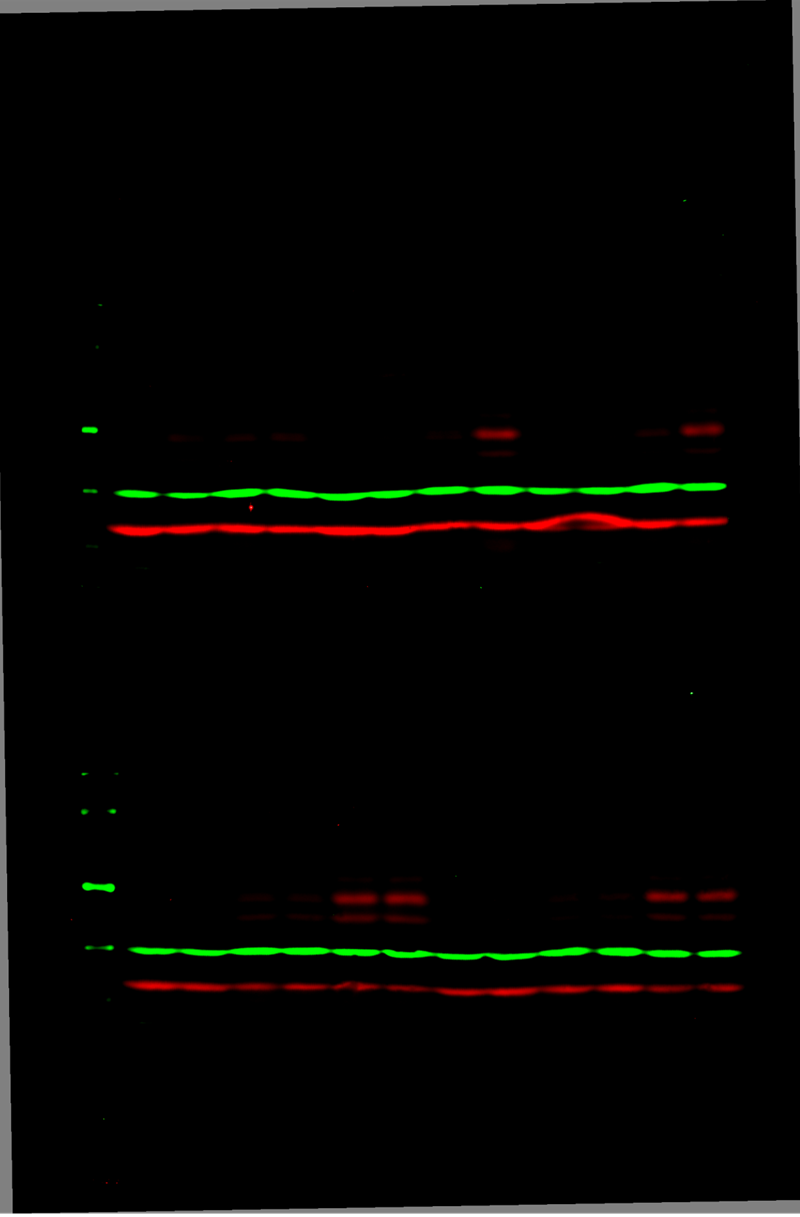

Supplement: Supplementary file 1 [file DataSheet_1.zip › WB/28-p38-KO-down.tif]

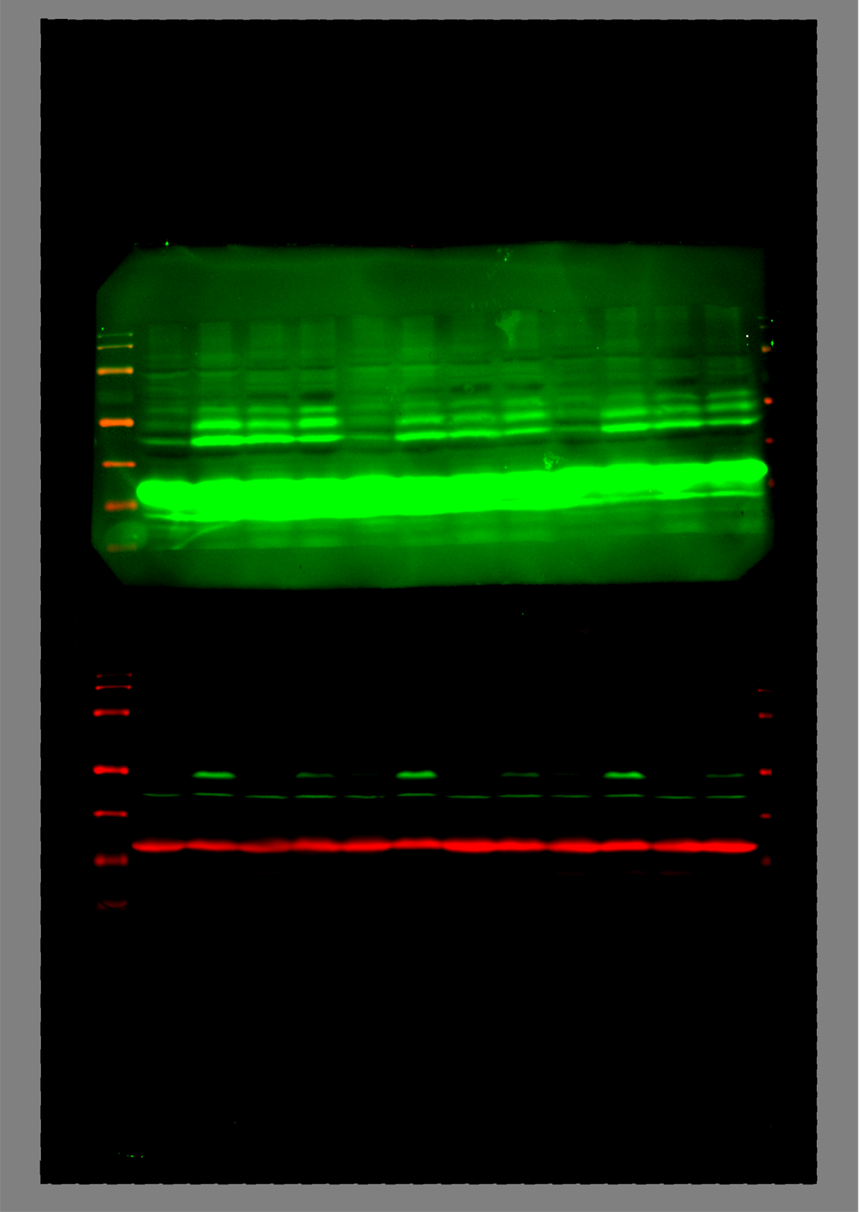

Supplement: Supplementary file 1 [file DataSheet_1.zip › WB/29-JNK-KO-down.tif]

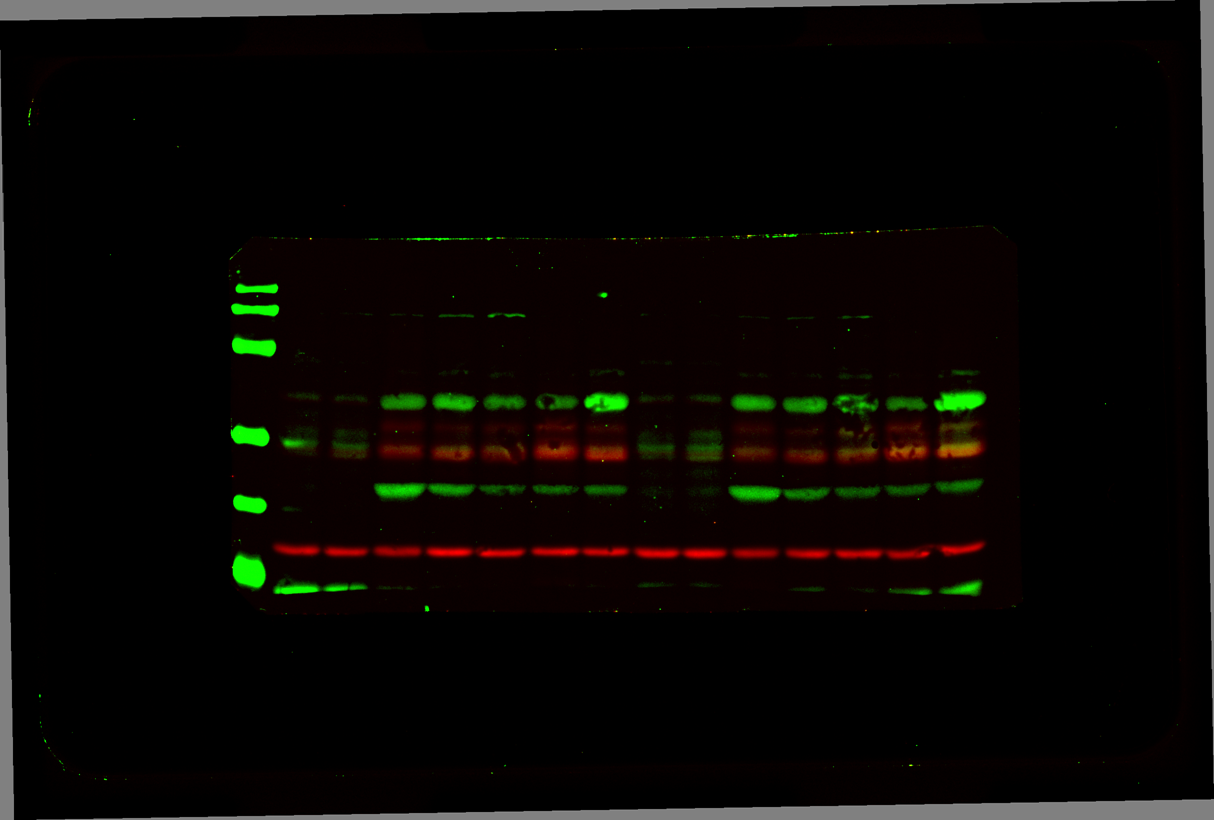

Supplement: Supplementary file 1 [file DataSheet_1.zip › WB/2-SMA2.tif]

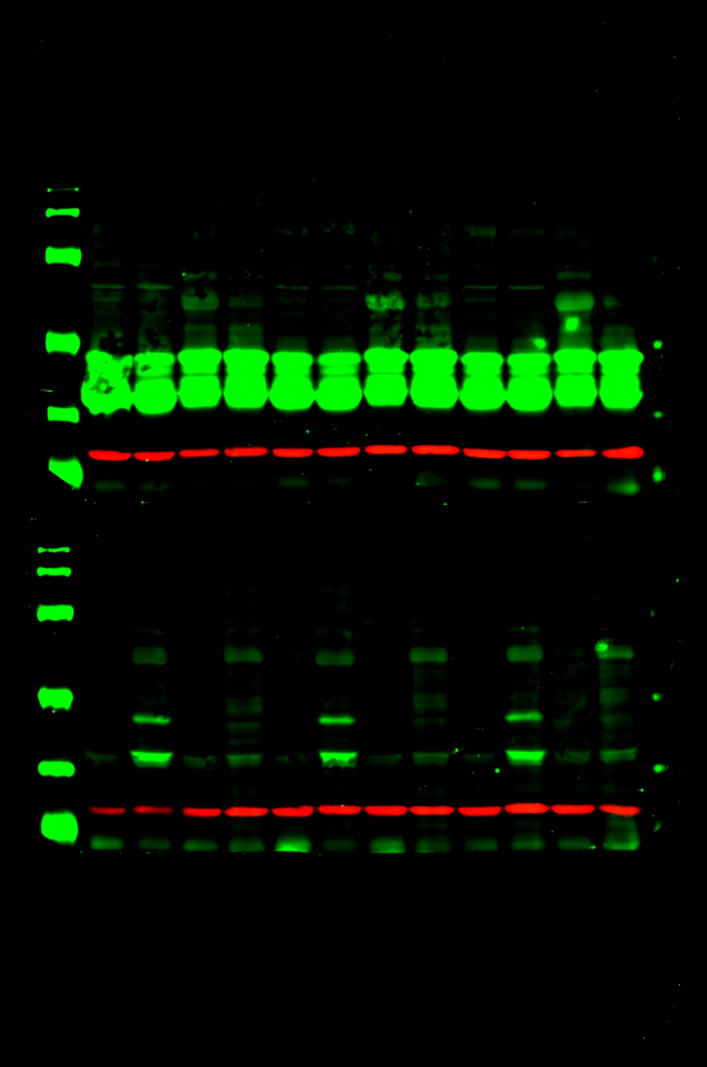

Supplement: Supplementary file 1 [file DataSheet_1.zip › WB/30-pJNK-KO-down.tif]

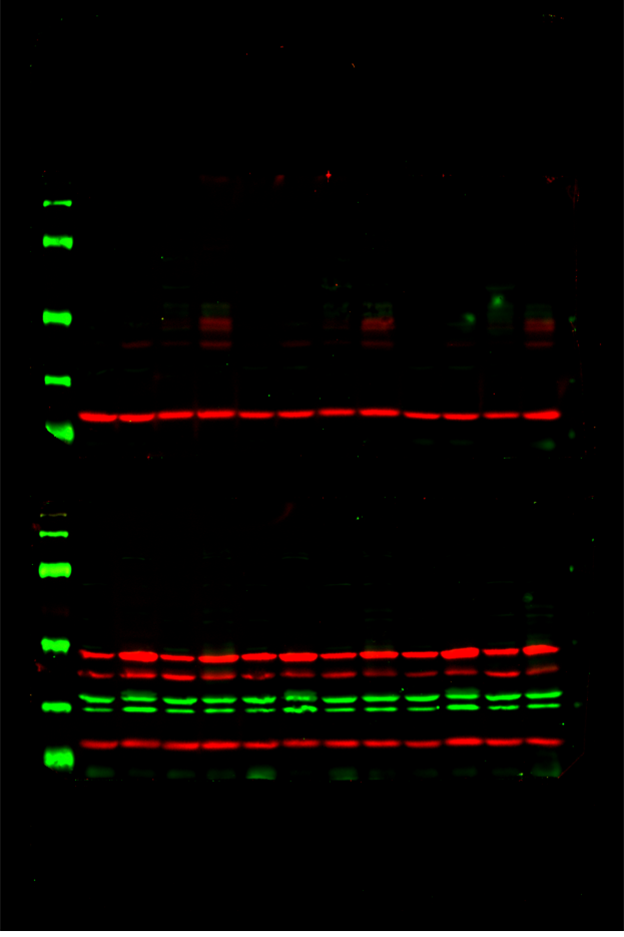

Supplement: Supplementary file 1 [file DataSheet_1.zip › WB/31-ERK-KO-down.tif]

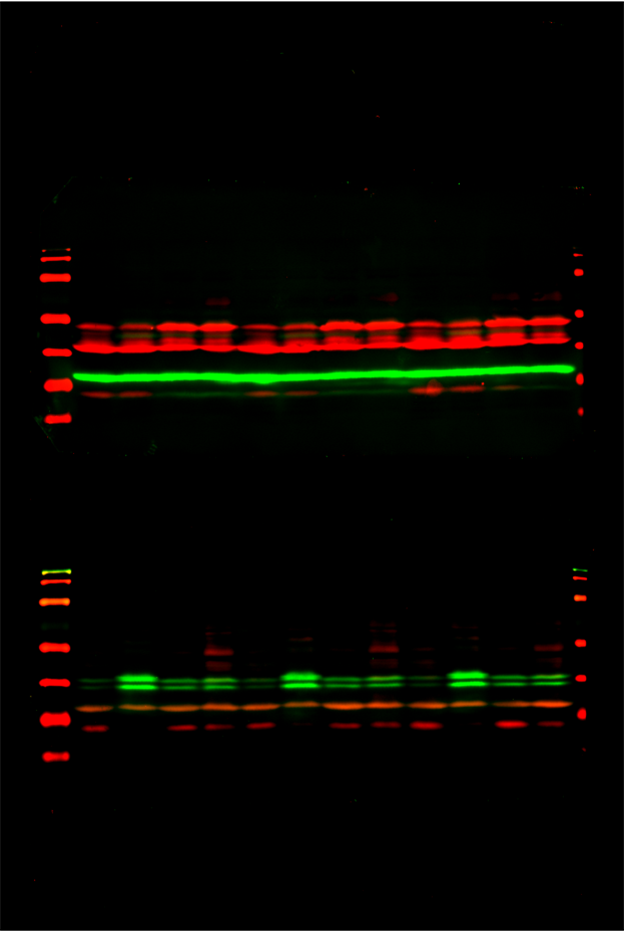

Supplement: Supplementary file 1 [file DataSheet_1.zip › WB/32-pERK-KO-down.tif]

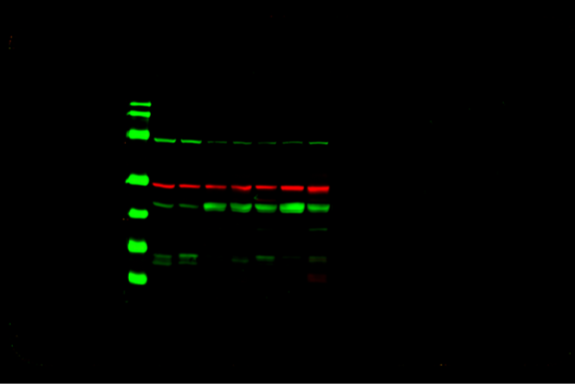

Supplement: Supplementary file 1 [file DataSheet_1.zip › WB/3-E-cadherin1.tif]

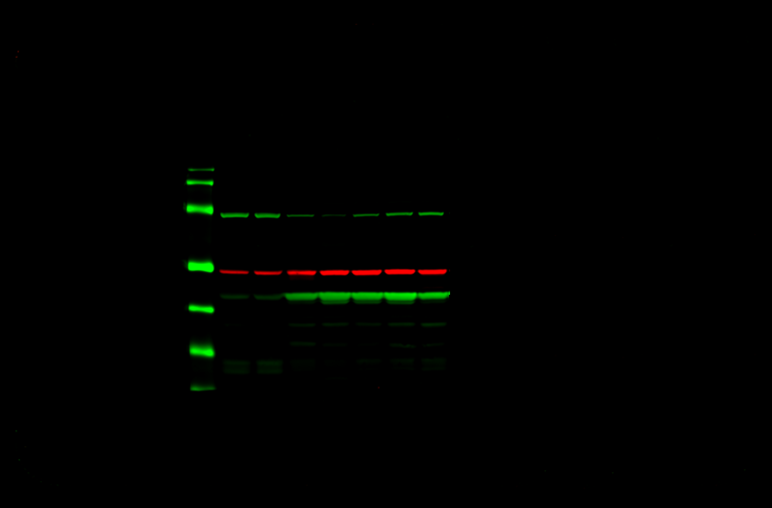

Supplement: Supplementary file 1 [file DataSheet_1.zip › WB/4-E-cadherin2.tif]

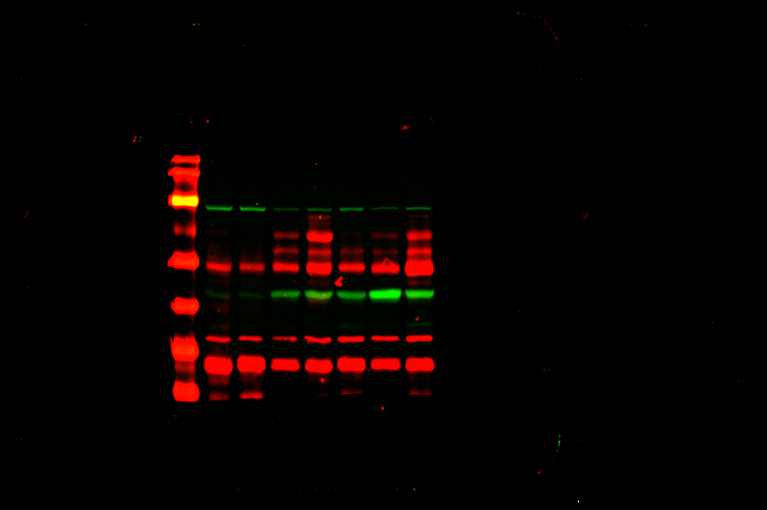

Supplement: Supplementary file 1 [file DataSheet_1.zip › WB/5-E-cadherin3.tif]

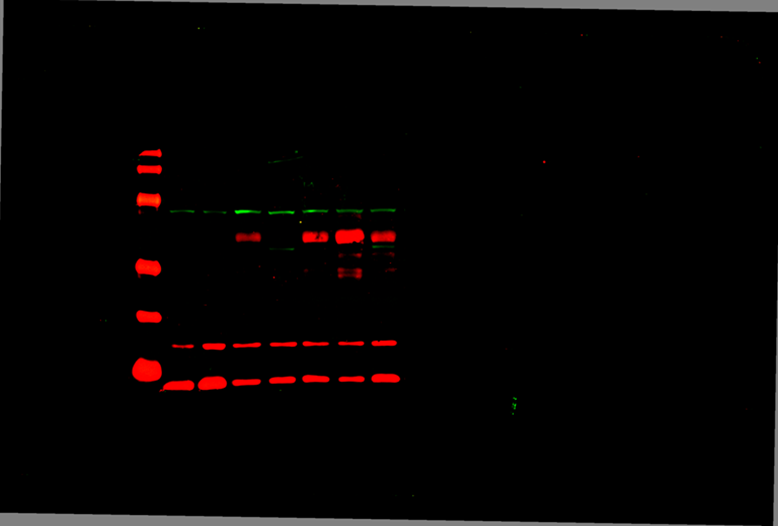

Supplement: Supplementary file 1 [file DataSheet_1.zip › WB/6-TRPC6-1.tif]

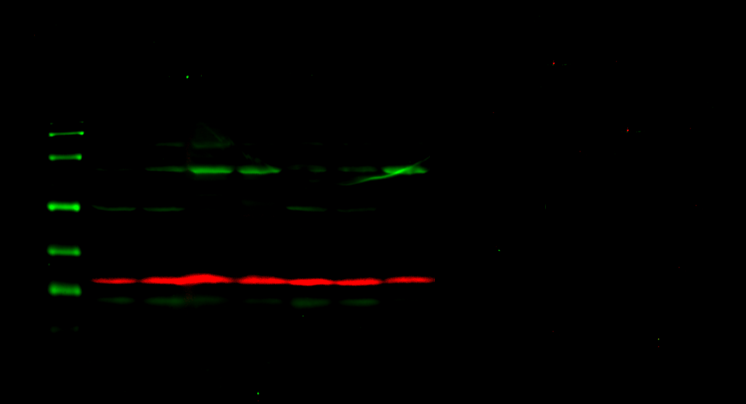

Supplement: Supplementary file 1 [file DataSheet_1.zip › WB/7-TRPC6-2.tif]

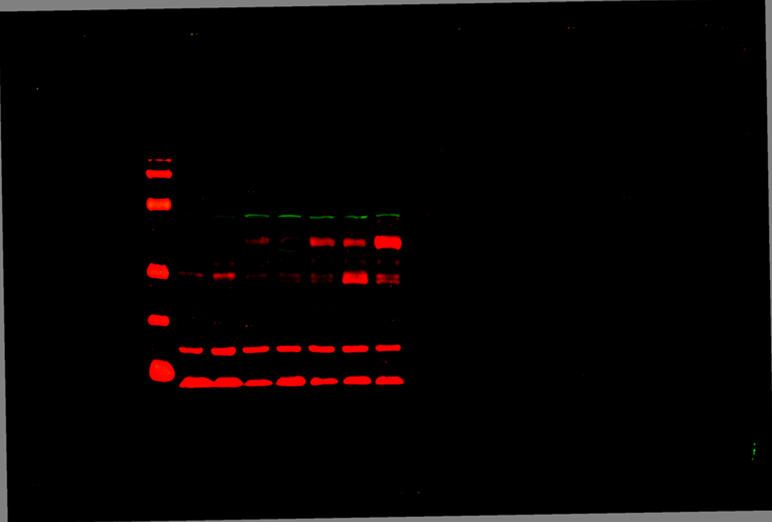

Supplement: Supplementary file 1 [file DataSheet_1.zip › WB/8-TRPC6-3.tif]

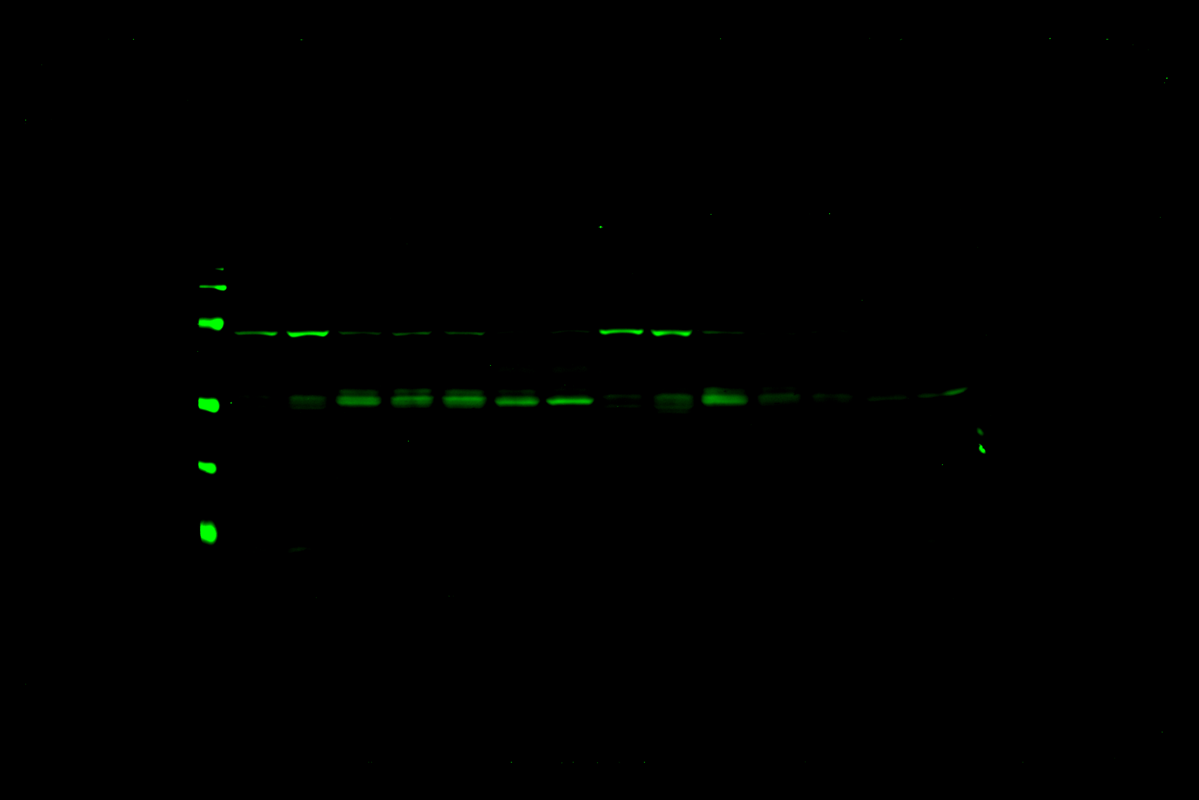

Supplement: Supplementary file 1 [file DataSheet_1.zip › WB/9-CnA.tif]
