# Supplementary material for: Huangkui Capsule Ameliorates Renal Fibrosis in a Unilateral Ureteral Obstruction Mouse Model Through TRPC6 Dependent Signaling Pathways
Source: Front Pharmacol. 2020 Jul 3;11:996. doi: 10.3389/fphar.2020.00996 (PMC7350529; doi:10.3389/fphar.2020.00996)
Supplement: Supplementary file 2 [file DataSheet_2.docx]

**Huangkui Capsule Ameliorates Renal Fibrosis in a Unilateral Ureteral Obstruction Mouse Model through TRPC6 Dependent Signaling Pathway**

**Supplementary Tables**

**Table S1** Oligonucleotide primers used for qRT-PCR in this study

| Target gene | Forward primers (5′– 3′) | Reverse primer (5′–3′) |
| --- | --- | --- |
| *Mcp-1* | ATTGGGATCATCTTGCTGGT | CCTGCTGTTCACAGTTGCC |
| *Vcam-1* | TCTCTCAGGAAATGCCACCC | CACAGCCAATAGCAGCACAC |
| *Il-1β* | CTTCAGGCAGGCAGTATCACTCAT | TCTAATGGGAACGTCACACACCAG |
| *Il-12* | CTTCTTCATCAGGGACATCATC | CCTCCTCTGTCTCCTTCATCTT |
| *Il-6* | TGATGGGTGTGAACCACGAG | GCCCTTCCACAATGCCAAAG |
| *Mmp-12* | CACAACAGTGGGAGAGAAAA | AGCTTGAATACCAGATGGGATG |
| *Tgf-β* | CTCCCGTGGCTTCTAGTGC | GCCTTAGTTTGGACAGGATCTG |
| *α-Sma* | GTTCAGTGGTGCCTCTGTCA | ACTGGGACGACATGGAAAAG |
| *E-cadherin* | ACAGCCCCGCCTTATGATT | TCGGAACCGCTTCCTTCA |
| *Gapdh* | CAGGGCTGCCTTCTCTTGTG | GATGGTGATGGGTTTCCCGT |

**Table S2** Effect of HKC on the levels of Scr, BUN and hydroxyproline in UUO mice.

| Groups | Scr (μmol/L)  Mean ± SEM | BUN (mmol/L)  Mean ± SEM | Hydroxyproline(μg/L) Mean ± SEM |
| --- | --- | --- | --- |
| Sham | 10.25 ± 1.06 | 6.45 ± 0.17 | 14.33 ± 0.62 |
| HKC (1.5 g/kg) | 9.33 ± 0.61 | 6.52 ± 0.11 | 14.45 ± 0.60 |
| UUO | 10.22 ± 1.38 | 9.17 ± 0.23^##^ | 17.13 ± 0.37^##^ |
| UUO + HKC (0.15 mg/kg) | 10.08 ± 1.51 | 7.60 ± 0.30^**^ | 16.55 ± 0.68 |
| UUO + HKC (0.5 mg/kg) | 9.90 ± 0.72 | 7.44 ± 0.22^**^ | 15.61 ± 0.44 |
| UUO + HKC (1.5 mg/kg) | 9.23 ± 0.69 | 7.38 ± 0.19^**^ | 14.97 ± 0.57^*^ |
| UUO + Losartan | 11.14 ± 0.37 | 7.85 ± 0.19^**^ | 16.53 ± 0.42 |

^##^*P* < 0.01, UUO group *vs.* sham group; ^*^*P* < 0.05, ^**^*P* < 0.01, UUO + HKC group or UUO + losartan group *vs.* UUO group. Data represent means ± SEM (n = 10).

**Supplementary Figures**


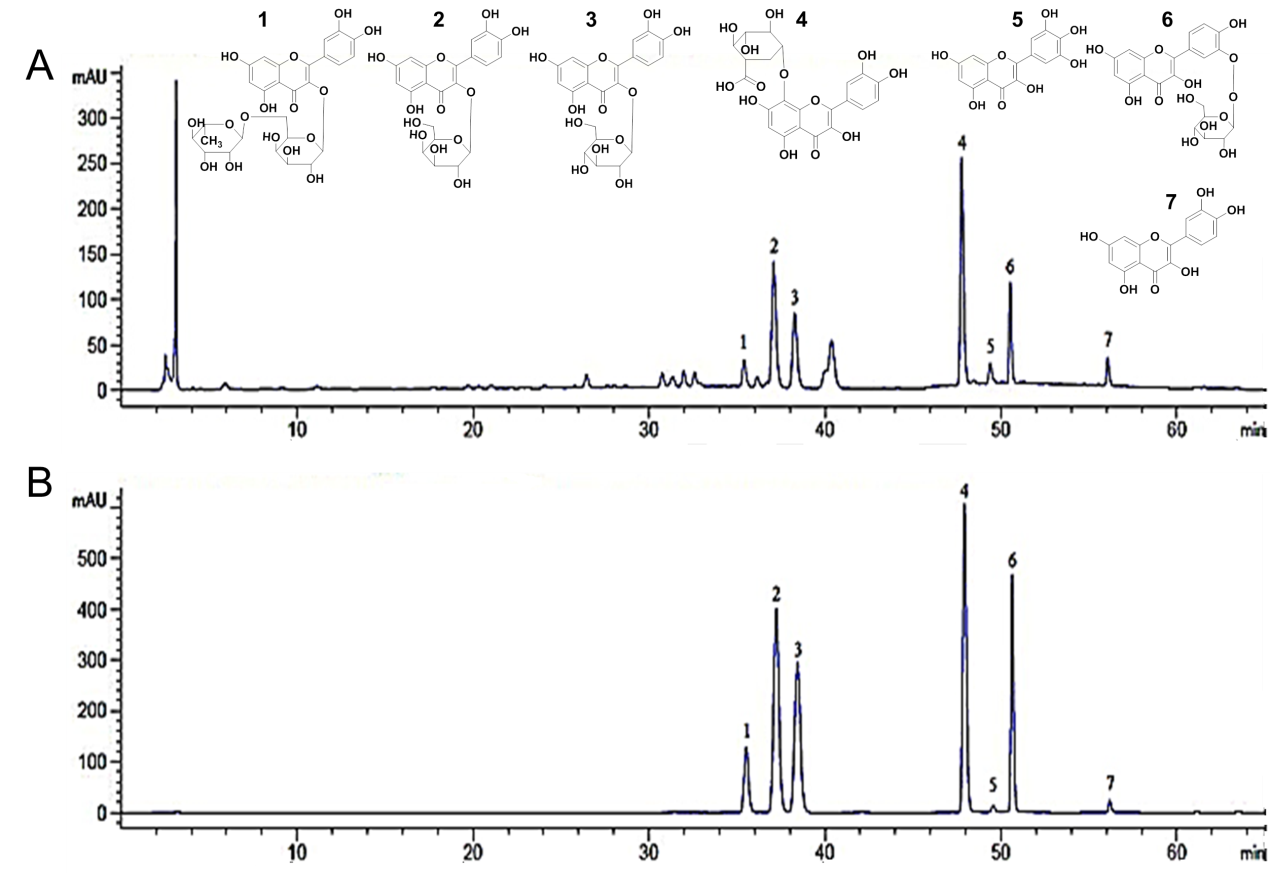


**Figure S1.** High performance liquid chromatography analysis of components in HKC. Representative HPLC fingerprints of HKC (A) and mixed standards (B). Detection wavelength was 254 nm. These compounds are quercetin-3-*O*-robinobioside (1), hyperoside (2), isoquercitrin (3), gossypetin-8-*O*-*β*-*D*-glucuropyranoside (4), myricetin (5), quercetin- 3′-*O*-*β*-*D*-glucoside (6) and quercetin (7).
